# Potential‐ and Time‐Dependent Operando X‐Ray Absorption Study of Cu_2_O Microcrystals Transformations during Nitrate Reduction to Ammonia

**DOI:** 10.1002/cssc.202501785

**Published:** 2025-10-14

**Authors:** Rizki Marcony Surya, Surya Pratap Singh, Kosuke Beppu, Fumiaki Amano

**Affiliations:** ^1^ Department of Applied Chemistry for Environment Tokyo Metropolitan University 1‐1 Minami‐Osawa Tokyo 192‐0397 Hachioji Japan

**Keywords:** cuprous oxide, electrocatalysis, nitrate reduction reaction, operando X‐ray absorption fine structure, oxide‐derived copper

## Abstract

Electrochemical nitrate reduction reaction (NO_3_RR) represents a sustainable, carbon‐neutral alternative to the Haber–Bosch process for ammonia synthesis. Time‐resolved operando X‐ray absorption spectroscopy reveals the chemical states and structural evolution of copper(I) oxide microcrystals deposited on carbon fiber (Cu_2_O/C) across a potential range of +0.6 to −0.7 V versus reversible hydrogen electrode (V_RHE_), where nitrate reduction to nitrite and ammonia occurs. Without nitrate, Cu_2_O microcrystals are quickly reduced to metallic Cu(0) aggregates at low reduction potentials (≈0.1 V_RHE_). In contrast, only 29% Cu(0) is observed in 0.1 m NaNO_3_ at 0.1 V_RHE_, indicating that nitrate adsorption passivates the surface and promotes selective electron transfer to nitrate, thereby retarding the kinetics of Cu_2_O microcrystals transformation to Cu(0) particles. Ammonia formation initiates at −0.3 V_RHE_ in 0.1 m NaNO_3_ (pH 13) solution, accompanied by the formation of metallic copper particles for the hydrogenation of the intermediates. The Faradaic efficiency (FE) of ammonia is increased with more negative potential, accompanied by the formation of metallic Cu(0) particles. The fully reduced Cu particles exhibit superior NO_3_RR activity to produce nitrite at lower reduction potentials and ammonia at higher reduction potentials, achieving 89.7% ammonia FE at −0.7 V_RHE_.

## Graphical abstract

Operando quick X‐ray absorption spectroscopy of electrodeposited Cu_2_O microcrystals on carbon fibers reveals time‐resolved Cu(I)‐to‐Cu(0) transformation and metallic bond formation over +0.6 to −0.7 V versus RHE with 0.1 V intervals, correlating the oxidation state and structure with the activity and selectivity into ammonia and nitrite during nitrate reduction reaction (NO_3_RR).

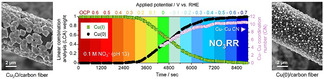

## 1 Introduction

Ammonia (NH_3_), or its protonated form, ammonium (NH_4_^+^), is a valuable carbon‐free fuel, hydrogen carrier, and fertilizer precursor. The Haber–Bosch process dominates NH_3_ production, operating at high temperatures and pressures (above 400 °C and 200 bar). This energy‐intensive method accounts for ≈1.4% of global CO_2_ emissions,^[^ ^1^, ^2^ ^]^ and potentially exceeds 1,300 Mt‐CO_2_eq annually by 2050.^[^ ^3^ ^]^ As a greener alternative, electrochemical nitrate reduction reaction (NO_3_RR) enables sustainable NH_3_ synthesis under ambient conditions while mitigating NO_3_^−^ pollution.^[^ ^1^, ^2^, ^4^ ^]^ Various strategies have been explored to enhance NO_3_^−^‐to‐NH_3_ conversion, including mechanistic investigations on transition‐metal catalysts, proton regeneration, and anode‐assisted reactions such as formic acid and sulfur ion electrooxidation.^[^ ^5^, ^6^, ^7^, ^8^ ^]^

Copper and its oxides are among the most effective electrocatalysts for NO_3_RR to NH_3_ (or NH_4_^+^ in water), achieving high Faradaic efficiencies (FEs) (72–99%) with substantial production rates (Table S1, Supporting Information).^[^ ^9^, ^10^, ^11^, ^12^, ^13^, ^14^, ^15^, ^16^, ^17^, ^18^, ^19^, ^20^, ^21^, ^22^, ^23^ ^]^ The standard potential for NO_3_^−^ + 6H_2_O + 8e^−^ → NH_3_ + 9OH^−^ (+0.67 V_RHE_) is lower than that for NO_3_^−^ + H_2_O + 2e^−^ → NO_2_^−^ + 2OH^−^ (+0.84 V_RHE_), often yielding nitrite as an intermediate. According to the Pourbaix diagram,^[^ ^24^ ^]^ Cu‐based catalysts undergo dynamic transitions between metallic and oxidized states, depending on the pH and applied potential. This behavior has sparked debates on its specific active site for high catalytic performance. Some works suggest the importance of Cu oxides and surface oxygen species,^[^ ^9^, ^11^, ^12^, ^14^, ^15^, ^25^ ^]^ while others argue that metallic Cu drives the performance with scarce involvement from the oxide.^[^ ^13^, ^18^, ^21^, ^26^, ^27^ ^]^ Recently, some teams linked two‐electron NO_3_‐to‐NO_2_^−^ activity to Cu/Cu_2_O interface, and oxide‐derived metallic Cu(0) enhanced NH_3_ yield and selectivity at higher reductive potentials.^[^ ^20^, ^22^, ^28^ ^]^ However, the real relationship between catalytic structure and the activity/selectivity of these oxide‐derived Cu species remains in disagreement since the unstable Cu(I) is electrochemically changed to Cu(0) even during NO_3_RR.

Operando X‐ray absorption spectroscopy (XAS) is a powerful in situ characterization tool for probing the oxidation states and structural evolution of electrocatalysts.^[^ ^29^, ^30^ ^]^ X‐ray absorption near‐edge structure (XANES) is sensitive to electronic states, while extended X‐ray absorption fine structure (EXAFS) reveals local coordination and bond distances.^[^ ^31^, ^32^, ^33^ ^]^ For NO_3_RR, an operando XAS study was employed for Cu_2_O nanocubes (30–40 nm) with Nafion ionomer as a binder in 8 mM NaNO_3_ (pH 12) at two potentials, 0.1 and −0.3 V_RHE_.^[^ ^20^ ^]^ Our study used Cu_2_O polycrystals (≈1 μm) deposited on carbon fibers without any binder, across the full potential range relevant to nitrate reduction, and in a more concentrated electrolyte, 100 mM NaNO_3_ with 0.1 m NaOH (pH 13). This configuration minimizes mass transport effects (e.g., nitrate depletion, pH drift), allowing clearer observation of interfacial processes. Under these conditions, we observe strong nitrate adsorption on the binder‐free Cu_2_O surface, which passivates surface Cu^+^ sites and delays their reduction to Cu^0^—a behavior not previously reported. This interaction promotes selective electron transfer toward NO_3_RR, yielding NO_2_^−^ and NH_4_^+^ with about 100% FE. In contrast, in a nitrate‐free solution, rapid Cu_2_O reduction to metallic Cu dominates.

Operando quick‐XAS was performed under stepwise chronoamperometry from 0.6 to −0.7 V_RHE_ with 0.1 V intervals to track the more detailed chemical and structural changes during NO_3_RR. The measured current density represents the activity of the Cu catalysts, and the reaction selectivity was evaluated by analyzing the electrolyte after the stepwise chronoamperometry. We also performed the potential‐ and time‐dependent operando XAS in the absence of NO_3_^−^ to understand the catalysis and the formation of metallic copper during the hydrogen evolution reaction (HER) in an alkaline environment. The working state of the copper species may not be uniform, and the heterogeneity may lead to discrepancies in interpretation. Therefore, the spent electrodes obtained using the same protocols were characterized by scanning electron microscopy (SEM) and X‐ray diffraction (XRD) to check morphology and crystalline phase. We have compared the catalytic properties with a fully reduced electrode to identify the effect of applied potential on the metallic Cu(0) surface. Our operando XAS‐based methodology provides the real relationship between catalytic structure and the activity/selectivity of binder‐less electrodeposited micrometer‐sized precatalysts while minimizing fluctuations in the local reaction environment at higher concentrations.

## 2 Results and Discussion

**Figure 1:**
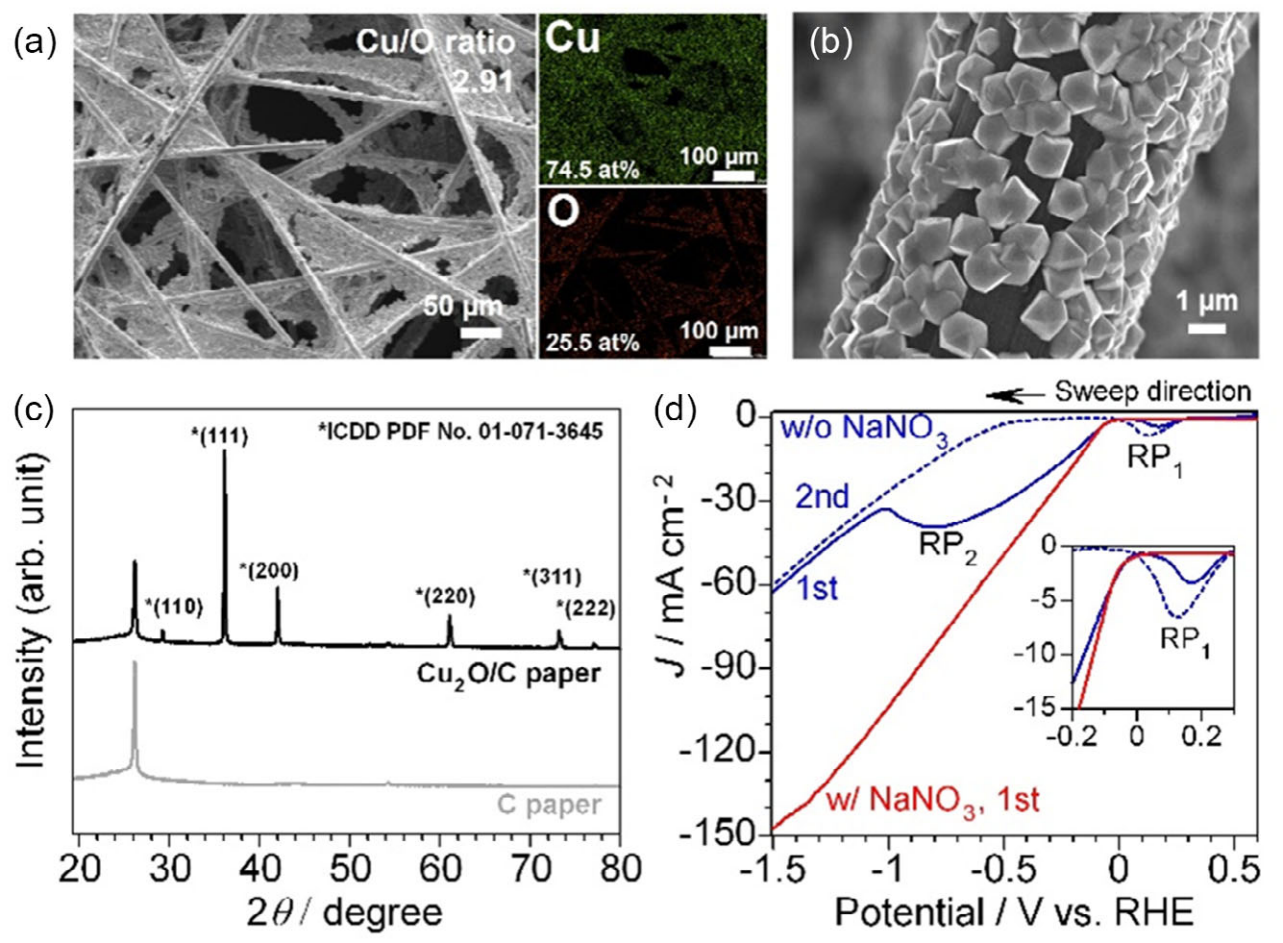
a,b) SEM–EDS images and c) XRD patterns of Cu_2_O/C electrode prepared by electrodeposition method. d) LSV curves in 0.1 m NaOH (pH 13) with and without 0.1 m NaNO_3_.

Cu_2_O (2.25 mg cm^−2^) was electrodeposited from copper(II) lactate complex in alkaline solution (pH 11) onto carbon fiber at 0.45 V_RHE_ with a charge density limit of −3.0 C cm^−2^ (Figure S1, Supporting Information).^[^ ^34^ ^]^ Energy‐dispersive X‐ray spectroscopy (EDS) analysis revealed that the Cu_2_O/C electrode consisted of 74.5% copper and 25.5% oxygen (**Figure** **1**a) in the form of polycrystalline grains (≈1 μm) with varied facet orientations (Figure 1b). Cu_2_O cube and octahedron exhibits {100} and {111} facets, respectively.^[^ ^35^, ^36^ ^]^ XRD patterns confirmed the presence of Cu_2_O phase (ICDD PDF No. 01‐071‐3645), consisting of (110), (111), (200), (220), (311), and (222) peaks (Figure 1c). Binder‐less Cu_2_O microcrystals were successfully prepared on carbon fibers by the electrodeposition method.

Linear sweep voltammetry (LSV) results in 0.1 m NaOH (pH 13) without NO_3_^−^ (Figure 1d) show the first reduction peak (RP_1_) around 0.1 V_RHE_ in the first sweep, attributed to the reduction of oxide or hydroxide surface.^[^ ^19^, ^37^ ^]^ The second reduction peak (RP_2_) appears only at the first sweep, representing the reduction of Cu_2_O‐to‐Cu(0).^[^ ^20^, ^37^ ^]^ In the second sweep, RP_1_ is observed again, and the onset of HER was −0.5 V_RHE_. In contrast, in 0.1 m NaNO_3_ with NaOH (pH 13), both RP_1_ and RP_2_ are absent, and NO_3_RR begins around −0.05 V_RHE_. The disappearance of RP_2_ likely results from the overlap between Cu_2_O reduction and the onset of NO_3_RR. The absence of RP_1_ is attributed to preferential nitrate adsorption on the Cu_2_O/Cu surface,^[^ ^34^ ^]^ which may either passivate Cu^+^/Cu^0^ sites or divert them directly into the NO_3_RR pathway.

**Figure 2:**
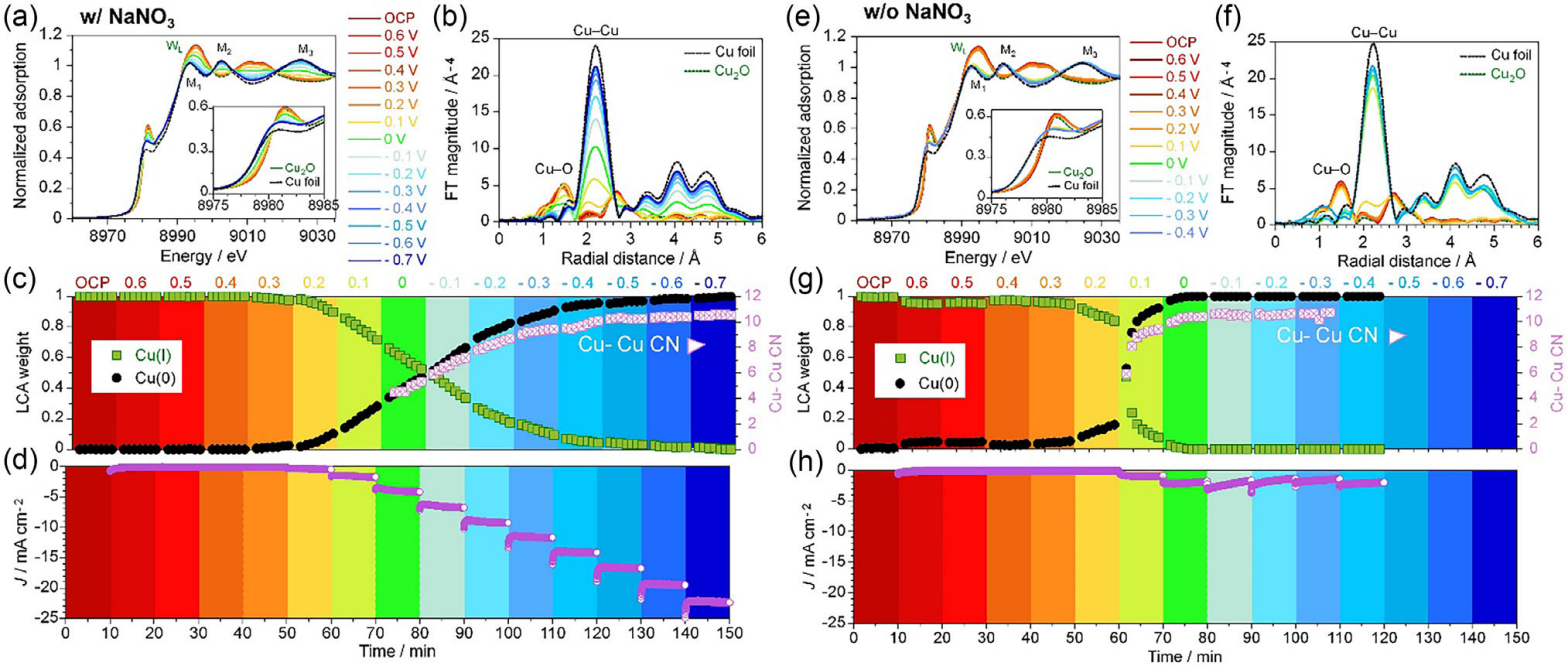
Operando quick‐XAS data for Cu_2_O/C electrode during NO_3_RR in 0.1 m NaNO_3_ at pH 13, stepping from +0.6 to −0.7 V_RHE_ in 600 s and 0.1 V intervals: a) normalized Cu K‐edge XANES spectra, b) Fourier‐transformed EXAFS spectra with reliable fitting (*R*‐factor: ranged from 0.001 to 0.005), c) LCA results (*R*‐factor: typically 10^−5^ to 10^−5^) from XANES and Cu—Cu CN from EXAFS curve fitting, and d) Current density–time profile during the in situ XAS measurement. e–h) Corresponding operando XAS data and current density profile of Cu_2_O/C in 0.1 m NaOH without NaNO_3_. Fitting details are in the Supporting Information (Table S2–S3, Supporting Information).

Operando quick‐XAS was employed at open circuit potential (OCP) and across the entire NO_3_RR potential range (+0.6–−0.7 V_RHE_) in 0.1 m NaNO_3_ with NaOH (pH 13) (**Figure** **2**a). At each potential with 0.1 V steps, seven spectra were collected over 10 min in quick‐scan transmission mode while simultaneously recording current density using a custom‐made H‐type cell (Figure S2, Supporting Information). The pH change was negligible during the stepwise potential sequence. The Cu K‐edge XANES of Cu_2_O/C at OCP closely resembled that of commercial Cu_2_O particles. From OCP to 0.4 V_RHE_, the XANES shape remained identical but gradually changed from 0.3 to −0.6 V_RHE_, resembling that of Cu foil at −0.7 V_RHE_, indicating a reduction to Cu(0). Slight deviations from the Cu foil spectrum are likely due to the smaller crystalline size.^[^ ^38^ ^]^ The XANES edge position reflects the oxidation state, with Cu(0) exhibiting a lower energy edge than Cu(I). The intensity and edge shifts (Figure 2a, inset) signify the coordination change from linear Cu(I) to face‐centered cubic Cu(0).^[^ ^32^, ^33^ ^]^ An intense feature at 8995 eV (*W*_L_) observed in the Cu_2_O represents transitions of core electrons (1*s* orbital) to vacant d‐orbitals. This *W*_L_ is less pronounced for pure metals due to dipole‐forbidden transitions at the K‐edge.^[^ ^31^, ^33^ ^]^ Metallic Cu is also characterized by a distinct doublet (M_1_, M_2_) and an M_3_ peak.^[^ ^39^ ^]^ During NO_3_RR, EXAFS oscillations (Figure S3, Supporting Information) changed with XANES, indicating concurrent electronic and structural transformation. Fourier‐transformed EXAFS spectra (Figure 2b) show the gradual decrease in the peaks around 1.5 Å (Cu—O) and 2.7 Å (Cu—(O)—Cu) of Cu_2_O at less than 0.4 V_RHE_. The Cu—Cu peaks of the metallic Cu appeared at 2.2 Å and in the 3.0–5.5 Å range at 0.1 V_RHE_, and gradually intensified as the potential moved to more negative values, as previously reported.^[^ ^20^, ^31^, ^40^, ^41^ ^]^

The XANES data were analyzed by linear combination fitting to quantify the Cu(I) and Cu(0) ratio (Figure 2c). Cu_2_O/C remained predominantly in the Cu(I) state for the initial 40 min (from OCP to 0.4 V_RHE_). The onset potential of cathodic current was 0.3 V_RHE_ for Cu_2_O/C, and the linear combination analysis (LCA) indicates a decrease in Cu(I) ratio and the formation of Cu(0). As the potential moves in a negative direction, the cathode current density increased, and the LCA weight of Cu(I) was gradually decreased, implying the partial reduction of Cu_2_O to Cu(0) (Figure 2d). At 0.0 V_RHE_, which is near the onset of NO_3_RR in LSV (Figure 1d), the Cu(0) weight reached 0.48 after 10 min, indicating that Cu_2_O was partially reduced before NO_3_RR. The kinetics of Cu(I)‐to‐Cu(0) reduction were almost constant at the potential range from 0.1 to −0.1 V_RHE_, suggesting the potential‐independent rate‐determining step. The rate of LCA weight change was 0.02 per minute. The gradual reduction to Cu(0) continued at around −0.5 V_RHE_, showing the sluggish kinetics into fully reduced Cu(0).

The Cu—Cu coordination number (CN) was determined by EXAFS curve fitting in the first coordination sphere of metallic copper (Figure 2c, right axis). The fitting was limited to potentials over 0 V_RHE_ due to the second‐shell contributions from Cu_2_O at anodic potentials. The Cu—Cu CN was 4.5 at 0.0 V_RHE_, increasing to 5.3 after 10 min. An increase in cathodic potential gradually raises the Cu—Cu CN to 10.5, consistent with the increasing trend in Cu(0) LCA weight. Even after complete reduction, the CN remained below that of bulk Cu foil (CN = 12), indicating smaller crystalline sizes, a few nanometers.^[^ ^38^ ^]^

To investigate the impact of NO_3_^−^ on the reduction behavior, we performed the potential and time‐dependent XAS in a nitrate‐free 0.1 m NaOH solution. We found that the reduction behavior of Cu_2_O/C in the absence of NO_3_^−^ was significantly different from that during NO_3_RR. The operando XAS analysis (Figure 2e–h) showed a rapid reduction from Cu(I) to Cu(0) at more positive potentials. The rate of LCA weight change was 0.01 and 0.25 per minute at 0.0 and 0.1 V_RHE_, respectively. The reduction of Cu(I) to Cu(0) was significantly accelerated at the onset potential. The Cu(0) LCA weight reached 0.93 at 0.1 V_RHE_ after 10 min, and the Cu—Cu CN was 9.4, suggesting the metallic cluster formation. Cu_2_O/C is confirmed to be fully reduced to the metallic state (CN > 10.6) at 0.0 V_RHE_. The XAS spectra were unchanged until the potential reached −0.3 V_RHE_. At more negative potentials, the bubble of H_2_ interferes with the X‐ray due to HER, but the current density was small due to the lower HER activity of the metallic copper surface. Density functional theory (DFT) calculation has predicted that NO_3_RR is more selective on the Cu surface compared with HER.^[^ ^42^ ^]^ In the nitrate‐containing solution, the higher current densities accounted for efficient electron transfer to the adsorbed NO_3_^−^. On the other hand, a small reductive current density was sufficient to reduce Cu_2_O to Cu(0) between 0.2 and 0.0 V_RHE_ in nitrate‐free conditions. The Cu(I)‐to‐Cu(0) reduction kinetics in 0.1 m NaOH (0.25 min^−1^) were ten times higher than those in 0.1 m NaNO_3_ (0.02 min^−1^), suggesting that NO_3_RR suppresses the self‐reduction of Cu_2_O. NO_3_^−^ is adsorbed on the Cu_2_O surface, passivating it by promoting preferential electron transfer to NO_3_^−^ over Cu(I), thereby hindering the direct reduction of Cu_2_O.^[^ ^34^ ^]^

**Figure 3:**
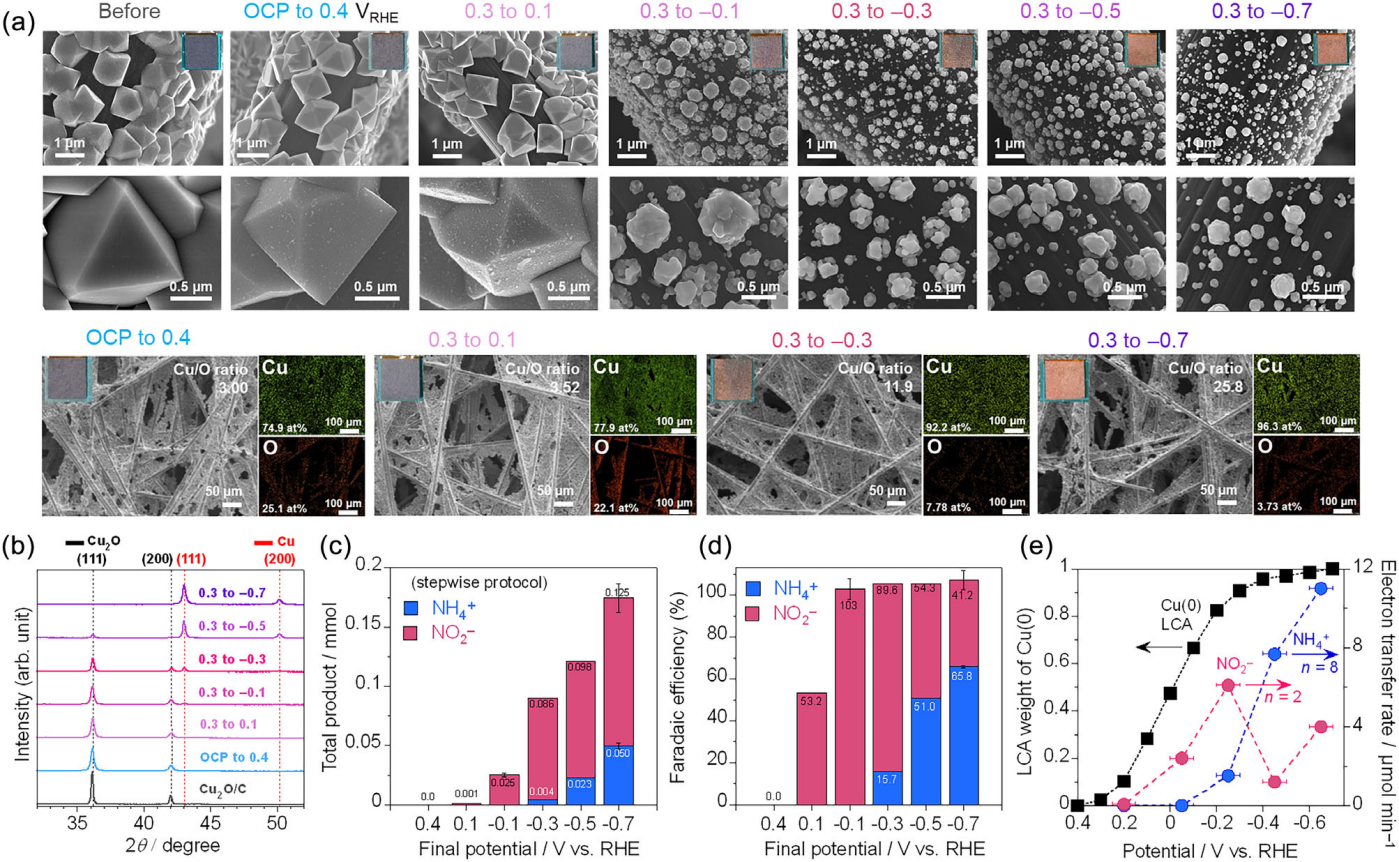
Characterization of Cu_2_O/C after the NO_3_RR at stepwise potential sequences, which are identical to the operando quick‐XAS measurement conditions and their product distribution: a) SEM images at high magnifications, photographs, and EDS mapping at low magnifications after the sequences at different final potentials, b) their XRD patterns, c) production amount of ammonia and nitrite determined after the potential sequences, d) FEs of each product calculated from the current densities during the potential sequences, and e) relationship between the Cu(0) weight determined by operando XANES analysis and the electron transfer rate during the 0.2 V potential step. Error bars are determined from three independent trials (Figure S7, Supporting Information).

Morphology and crystalline phase changes during operando XAS measurements were assessed ex situ across the sequences of applied potentials. The current density profiles recorded during the stepwise potential protocol closely matched those from in situ XAFS measurements (Figure S4, Supporting Information), indicating minimal effects from synchrotron radiation on the electrochemical reactions. SEM analysis (**Figure** **3**a and S5, Supporting Information) showed that the faceted shape of the Cu_2_O microcrystals remained unchanged from OCP to 0.4 V_RHE_. However, nanosized particles began to form on the Cu_2_O surface, as notably observed at 0.1 V_RHE_. A reductive potential step to −0.1 V_RHE_ disrupted the polycrystals, restructuring them into smaller submicron particles. The size of the particles was further reduced to 0.1–0.4 μm at −0.7 V_RHE_. EDS analysis revealed a marked increase in the Cu/O ratio from 3.0 to 25.8 during the transformation. XRD patterns (Figure 3b, magnified from Figure S6, Supporting Information) also showed a gradual decrease in Cu_2_O (111) and (200) peaks and a concurrent growth of Cu (111) and (200) peaks. The Cu(111) peak emerged at −0.1 V_RHE_, where a significant morphological transformation occurred. Further reduction led to the disappearance of Cu_2_O peaks. These ex situ data obtained using the same protocol were not only in line with the operando XAS result but also provided microstructural information, especially regarding heterogeneity. Microscopic analysis by SEM‐EDS is a valuable method interplaying with the “bulk” characterizations like XAS and XRD.

Electrocatalytic reaction products after the potential steps were quantified by ion chromatography (Figure S8, Supporting Information). No NH_4_^+^ or NO_2_^−^ was detected from OCP to 0.4 V_RHE_, as also indicated by the negligible current densities. Between 0.3 and 0.1 V_RHE_ (in 10 min steps of 0.1 V), minor NO_2_^−^ formation (≈1 μmol, 53.2% FE) was observed (Figure 3c,d, Table S4, Supporting Information). A potential step to −0.1 V_RHE_, where Cu_2_O microcrystals transformed into Cu_2_O—Cu mixed submicron particles, significantly boosted NO_2_^−^ production (25 μmol cm^−2^, ≈100% FE). At −0.3 V_RHE_, both NH_4_^+^ (15.7% FE) and NO_2_^−^ (89.6% FE) were produced, correlating with the formation of smaller Cu particles. Further reductions to metallic copper at −0.7 V_RHE_ increased NO_3_RR activity (−22 mA cm^−2^) and NH_4_^+^ yield to 51 μmol cm^−2^ (65.9% FE during the potential sequences). Here, we evaluated the electron transfer rate (Figure 3e), which is the product of each production yield during each 0.2 V step and electron stoichiometry (2 for NO_2_^−^ and 8 for NH_4_^+^). The electron transfer rate is a crucial factor in determining the kinetics of the electrocatalytic reactions at a given potential range. Electron consumption for nitrite formation emerged with the formation of Cu(0) and peaked at −0.2 V_RHE_, where Cu_2_O is still present. Ammonia formation dominated total electron consumption at −0.7 V_RHE_, where metallic Cu was the main component. These results indicate that NO_2_^−^ can be formed even on the oxide‐rich surface, and the main working state for NH_4_^+^ production is metallic Cu(0). The intermediate NO_2_^−^ is further reduced at higher cathodic potentials.

**Figure 4:**
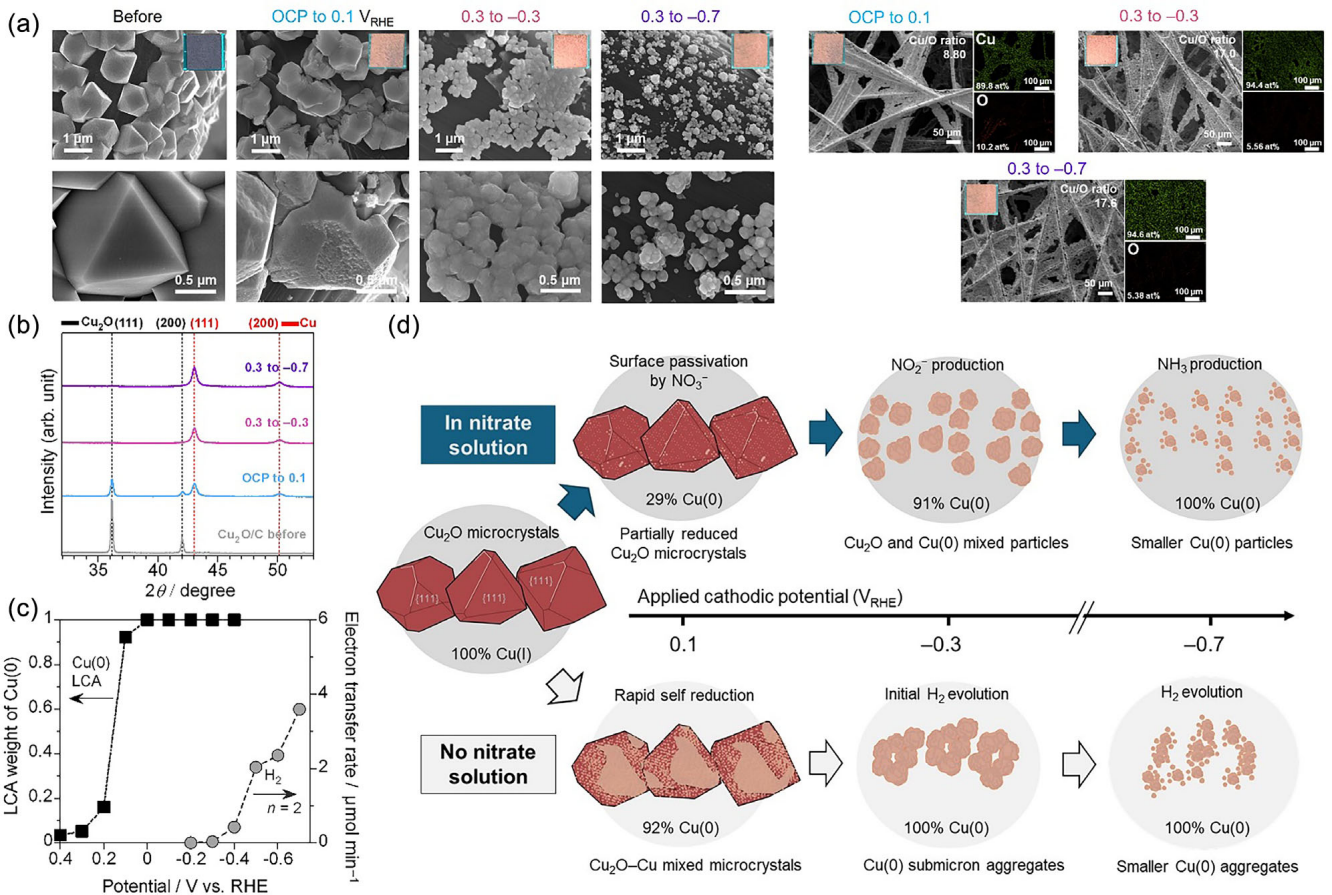
Characterization of Cu_2_O/C in 0.1 m NaOH without nitrate after stepwise potential sequences: a) SEM–EDS images after the sequences with different final potentials, b) their XRD patterns, c) relationship between the Cu(0) weight determined by operando XANES analysis and the electron transfer rate for HER (*n* = 2) quantified by gas chromatography, and d) illustration summarizing Cu_2_O transformation to metallic Cu particles under stepwise potentials.

In 0.1 m NaOH without NO_3_^−^, Cu_2_O polycrystals begin to degrade at more positive potentials (0.1 V_RHE_) than in the presence of nitrate, suggesting the passivation effect of NO_3_^−^ adsorbate. Nanoparticles formed within the polycrystalline matrix and exhibiting a significant increase in the Cu/O ratio (8.8) as confirmed by SEM–EDS analysis (**Figure** **4**a, S9, Supporting Information), suggesting the partial reduction. The SEM analysis revealed that, as the potential decreases to −0.3 and −0.7 V_RHE_, more extensive structural change occurs, leading to the formation of smaller aggregates. The dispersion of metallic copper species was lower than that observed during NO_3_RR. The lower dispersion would be attributed to the rapid reduction of Cu(I) to Cu(0) in the nitrate‐free electrolyte. XRD analysis (Figure 4b enlarged from Figure S10, Supporting Information) confirmed that metallic Cu (111) and (200) peaks appeared at −0.1 V_RHE_, while Cu_2_O reflections disappeared entirely by −0.3 V_RHE_. In SEM, the size of the aggregates decreased with higher cathodic potentials. The dynamic relocation process of metallic Cu species can be explained by the dissolution and redeposition during HER.^[^ ^17^, ^22^ ^]^ Figure 4c reveals that no H_2_ evolution detected at −0.1 V_RHE_ where all of Cu_2_O is reduced to Cu(0). Current density profiles (Figure S11, Supporting Information) confirm that the Cu(I) reduction is a faradaic process at a few mA cm^−2^. HER onset is observed around −0.3 V_RHE_, marked by a steady increase in current density and H_2_ evolution at higher reduction potentials. Metallic Cu can initiate water dissociation to produce adsorbed hydrogen species (i.e., Volmer step: H_2_O + e^−^ → OH^−^ + *H*_ad_). The *H*_ad_ generated on metallic Cu at potentials more negative than −0.3 V_RHE_ would play an important role in the hydrogenation of adsorbed NO_3_^−^ (*NO_3_) and NO_2_^−^ (*NO_2_) to ammonia.

Figure 4d summarizes the results of operando XAS analysis and ex situ characterization under stepwise potential sequences. At −0.1 V_RHE_, 29% of Cu_2_O was reduced to Cu(0) nanoparticles, even keeping the Cu_2_O microcrystal structure. At −0.3 V_RHE_, the simultaneous production of NH_4_^+^ (with nitrate) and H_2_ (without nitrate) suggests that metallic Cu is the primary hydrogenation site. Further Cu(I)‐to‐Cu(0) transformation during NO_3_RR forms smaller Cu(0) particles compared to the aggregates generated in nitrate‐free conditions. NH_4_^+^ FE increases at more reductive potentials over smaller Cu(0) particles. We propose that NO_3_^−^ adsorption promotes fast electron transfer while passivating the Cu_2_O surface, retarding Cu(I)‐to‐Cu(0) conversion both on the surface and in the bulk phase. Previous work suggests that Cu/Cu_2_O surfaces are kinetically stabilized by surface hydroxide layers, inferred from pH increases in neutral nitrate electrolytes.^[^ ^22^ ^]^ In contrast, our results show that in nitrate‐free alkaline solution, Cu_2_O microcrystals rapidly reduced to 92% Cu(0) before HER, forming Cu_2_O—Cu mixed microcrystals, and the kinetically fast reduction leads to the formation of submicron aggregates. The Cu(0) state was constant at higher reduction potentials, but the aggregates were relocated into more dispersed particles.

**Figure 5:**
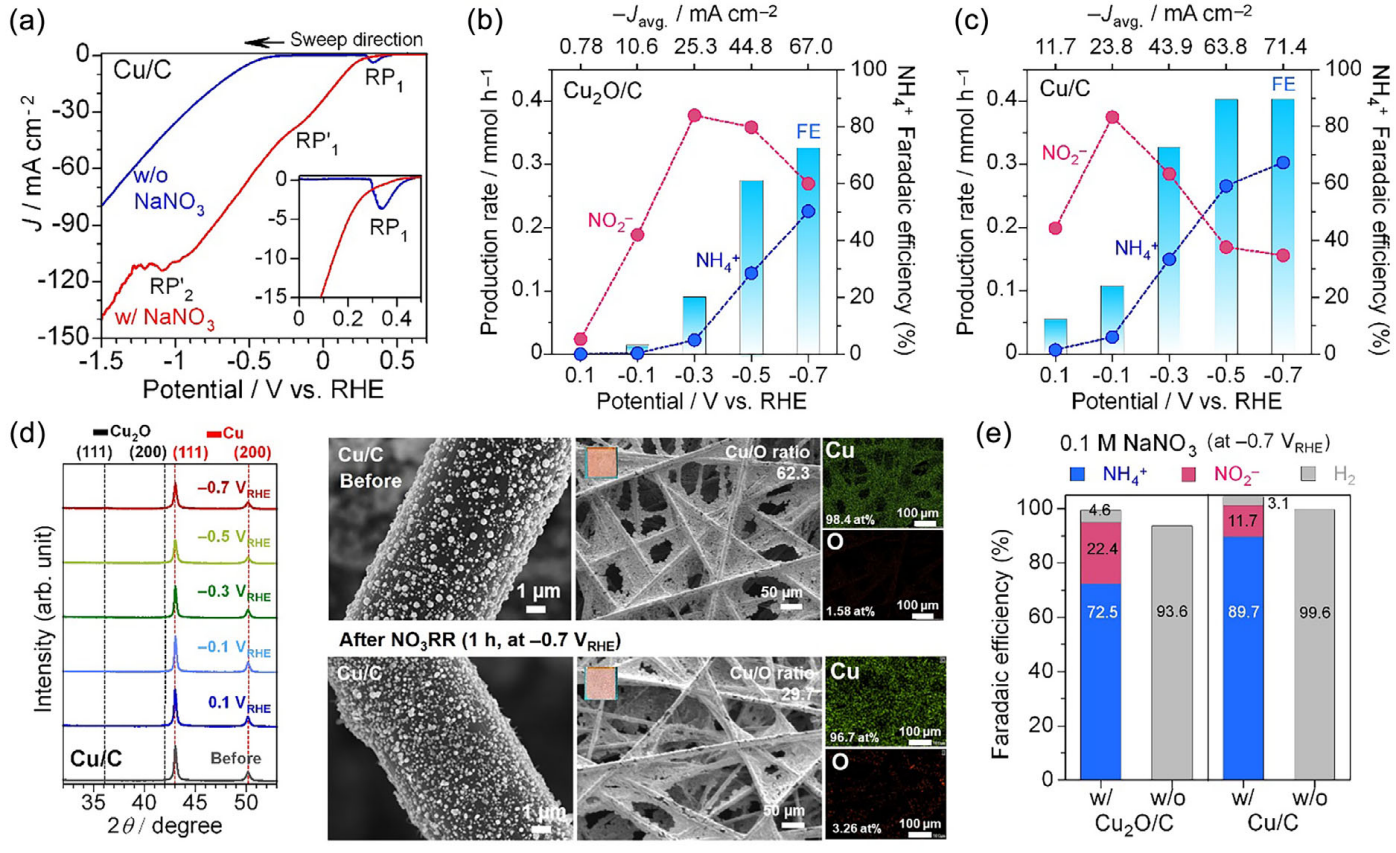
Comparison of Cu_2_O/C and electrochemically reduced Cu/C electrodes. a) LSV of Cu/C in 0.1 m NaOH with and without 0.1 m NaNO_3_. One‐hour NO_3_RR tests at static potentials of b) Cu_2_O/C and c) Cu/C electrodes, showing production rate and FE of NH_4_^+^. d) SEM–EDS and XRD characterizations of Cu/C before and after the NO_3_RR test at −0.7 V_RHE_. e) Impact of 0.1 m NaNO_3_ on product selectivity (NH_4_^+^, NO_2_^−^, and H_2_) at −0.7 V_RHE_. The corresponding current density–time plots, production rates, and FEs for all products are in (Figure S13, Supporting Information).

To better understand the role of Cu(I) and Cu(0) in NH_4_^+^ selectivity, a fully reduced Cu/C electrode was prepared by electroreduction of Cu_2_O/C in 0.1 m NaNO_3_ using the operando XAS protocol. A conventional H‐type cell was used to minimize ohmic losses, with stirring during the chronoamperometry test to enhance mass transport (Figure S12, Supporting Information). LSV of the oxide‐derived Cu/C electrode (**Figure** **5**a) shows RP_1_ in the absence of NO_3_^−^, suggesting the hydroxide formation on the surface in alkaline solution. The absence of RP_2_, which is assigned to Cu_2_O‐to‐Cu(0) reduction, confirms the absence of Cu_2_O. HER begins slightly earlier on the reduced Cu/C (−0.4 V_RHE_) than on the Cu_2_O/C electrode (−0.5 V_RHE_). RP_1_ also shifted to a more positive potential, suggesting the higher electrocatalytic activity. In 0.1 m NaNO_3_, RP_1_ was negligible due to the preferential adsorption of NO_3_^−^ on the Cu(0) surface. The reduced Cu/C showed a more positive onset (0.3 V_RHE_) for NO_3_RR compared to Cu_2_O/C (0 V_RHE_). The shoulder peaks at −0.1 V_RHE_ (RP’_1_) and −1.0 V_RHE_ (RP’_2_) have been assigned to NO_3_^−^‐to‐NO_2_^−^ conversion and subsequent reduction to NH_3_ and/or HER, respectively.^[^ ^17^, ^20^, ^37^ ^]^

Potentiostatic NO_3_RR results (Figure 5b,c) show higher current densities for the pre‐reduced Cu/C compared with Cu_2_O/C at all applied potentials, indicating superior catalytic activity of metallic Cu surfaces. The higher activity of Cu/C offers lower overpotentials, as confirmed by LSV profiles. The fully reduced Cu/C electrode generated NH_4_^+^ already at 0.1 V_RHE_ (6.8 μmol h^−1^). The ammonia production rate at −0.1 V_RHE_ (27 μmol h^−1^) was ≈12 times higher than that of Cu_2_O/C, where NH_4_^+^ begins forming with in situ Cu(0) formation. The NH_4_^+^ FE of Cu_2_O/C reaches only 72.5% even at −0.7 V_RHE_, while the reduced Cu/C achieves NH_4_^+^ FE of 89.4% at −0.5 V_RHE_ (see the details in Table S5–S6, Supporting Information). These results align with operando XAS findings, indicating that NO_3_RR to NO_2_^−^ dominates on Cu_2_O and Cu(0) surfaces at lower reduction potentials, and NH_4_^+^ formation is favored on metallic Cu(0) particles at more negative potentials. Smaller Cu(0) particles exhibit higher activity and selectivity for NO_3_RR to ammonia. The XRD patterns revealed no structural change in metallic Cu after the NO_3_RRs (Figure 5d). The Cu particle sizes decreased slightly from 0.1–0.4 to 0.1–0.3 μm after NO_3_RR at −0.7 V_RHE_, with similar ranges across all potentials (Figure S14, Supporting Information). In NO_3_RR, such behavior likely arises from the formation of copper‐ammine complexes, which facilitate redeposition and mitigate aggregation under reductive conditions.^[^ ^22^, ^43^, ^44^ ^]^ SEM‐EDS analysis showed a decrease in Cu/O ratio after the reaction, suggesting surface oxidation during NO_3_RR. The Cu/O ratio was increased at lower reduction potentials. Surface‐sensitive operando approaches could characterize such dynamic behaviors.

Figure 5e demonstrates that the adsorption of NO_3_^−^ dramatically suppresses H_2_ FE on Cu_2_O/C (4.6%) and Cu/C (3.1%) at −0.7 V_RHE_. In the absence of NO_3_^−^, Cu/C shows ≈100% H_2_ FE. The lower H_2_ FE for Cu_2_O/C resulted from electron consumption by Cu_2_O‐to‐Cu(0) reduction. Experimental and DFT studies show that Cu(I) sites bind *H*_ad_ weakly and have limited charge transfer capabilities, leading to high energy barriers for water activation and inhibiting the Volmer step relative to metallic Cu.^[^ ^45^ ^]^ NO_3_RR to NH_3_ on copper proceeds via two potential‐dependent steps. Initially, Cu(I) or Cu(0) surfaces promote the spontaneous formation of *NO_3_.^[^ ^20^, ^21^, ^34^ ^]^ At 0.1 to −0.1 V_RHE_, *NO_3_ is reduced to *NO_2_ when Cu(I) and Cu(0) coexist on the surface. At more reductive potentials (<−0.1 V_RHE_), NH_4_^+^ is formed as Cu(I) continues reducing to Cu(0). The metallic phase promotes the formation of *H*_ad_ and subsequent *NO_*x*_ hydrogenation due to its high electrical conductivity and lower energy barrier.^[^ ^20^, ^21^, ^42^ ^]^ The hydrogenation of *NO_2_ and *NH_2_OH is the rate‐determining steps for ammonia production.^[^ ^20^, ^21^ ^]^

The observed retardation in Cu(I)‐to‐Cu(0) transformation by nitrate adsorption reflects the strong surface interaction between NO_3_^−^ and Cu_2_O, which stabilizes the Cu(I) phase. However, once the surface is fully reduced, the resulting metallic Cu exhibits superior NO_3_RR activity, particularly for multi‐electron conversion to NH_4_^+^. Thus, Cu(I) acts as a transient state interacting with nitrate, while Cu(0) is the catalytically active phase for the selective reduction of nitrate to ammonia. This interpretation is supported by our operando XAS data, which show a gradual structural evolution accompanied by a shift in product selectivity. The complete transformation to Cu^0^ under cathodic bias is correlated with significantly enhanced NH_4_^+^ production, indicating that the smaller Cu^0^ particles serve as the catalytically active sites under NO_3_RR conditions relevant to ammonia generation.

## 3 Conclusion

Operando quick‐XAS was performed on Cu_2_O microcrystals, which were directly deposited on carbon fibers, under stepwise chronoamperometry in 100 mM NaNO_3_ (pH 13) from 0.6 to −0.7 V_RHE_ with 0.1 V intervals, with concurrent acquisition of current densities (tens of milliampere) and the ex situ selectivity analysis. The Cu_2_O was gradually reduced to metallic Cu particles, which enhanced NH_4_^+^ production, throughout several tens of minutes. In the absence of NaNO_3_, the reduction was completed at more positive potentials in a few minutes, suggesting that nitrate adsorption passivates the Cu_2_O surface during NO_3_RR. The coexistence of Cu(I) and metallic Cu particles, along with their heterogeneous structures, under working state contributed to the previous controversy in active site analysis for Cu_2_O‐derived electrocatalysts. Fully reduced Cu(0) particles with smaller sizes exhibit higher performance in NO_3_RR into nitrite at low reduction potential and ammonia at high reduction potentials compared with their oxide and in situ generated metallic Cu particles. These findings, based on the operando XAS approach, provide valuable insights for optimizing Cu‐based catalysts for selective electrochemical hydrogenation reactions, such as NO_3_RR to ammonia.

## Supporting Information

Experimental procedures, XAS fitting results, product analysis, Nyquist plots, current density–time plots, calibration curves, and SEM–EDS analysis after the reactions.

## Conflict of Interest

The authors declare no conflict of interest.

## Supporting information

Supplementary Material

## Data Availability

The data that support the findings of this study are available in the supplementary material of this article.
