# Supplementary material for: Potential‐ and Time‐Dependent Operando X‐Ray Absorption Study of Cu2O Microcrystals Transformations during Nitrate Reduction to Ammonia
Source: ChemSusChem. 2025 Oct 14;18(23):e202501785. doi: 10.1002/cssc.202501785 (PMC12665874; doi:10.1002/cssc.202501785)
Supplement: Supplementary file 1 — Supplementary Material [file CSSC-18-e202501785-s001.pdf]

Supporting information

Potential- and Time-Dependent Operando X-Ray Absorption Study of Cu_2O Microcrystals Transformations during Nitrate Reduction to Ammonia

*Rizki Marcony Surya, Surya Pratap Singh, Kosuke Beppu, and Fumiaki Amano**

Table of Contents

Experimental S3

Tables (Table S1–S6)...S6

Figures (Figure S1–S14)...S12

References S20

Experimental

Electrodeposition of Cu_2O onto carbon fiber (Cu_2O/C).

Unless otherwise noted, all reagents were obtained from Fujifilm Wako Pure Chemicals, Japan. A 3 cm × 3 cm carbon fiber substrate (GDL 29AA, SGL Carbon SIGRACET, Germany) with an aerial weight of 29 g m^{-2} and thickness of 180 μm was sequentially cleaned with ethanol and deionized water, dried, and treated with UV/ozone (Asumi Giken, Japan) for 2 minutes on each side (front and back) to enhance its hydrophilicity. It was masked with Kapton tape (Nilaco, Japan) to define a 2 cm × 3 cm area for electrodeposition.

Electrodeposition was conducted in a one-compartment, three-electrode cell using an Ag/AgCl reference electrode (+196 mV vs. standard hydrogen electrode, V_{SHE}), a Pt wire counter electrode, and the carbon fiber working electrode (Figure S1a). The electrolyte solution was a copper(II) lactate, composed of 0.2 M copper sulfate pentahydrate (99.9%) and 1.5 M D-lactic acid (85.0–92.0%) adjusted to pH 11 with 4 M NaOH (97.0%). The solution was stirred and maintained at 65°C in a water bath. Cu_2O was deposited at +0.45 V vs. reversible hydrogen electrode (V_{RHE}) using a potentiostat (SP-150e, BioLogic, France) with a charge density of 3.0 C cm^{-2} to control the loading amount. The resulting Cu_2O (2.25 mg cm^{-2})/C electrode was cut into 1 cm × 1 cm pieces for further use.

Electroreduction of Cu_2O/C to Cu/C.

Cu/C was prepared by electrochemically reducing Cu_2O/C using the same potential steps from +0.3 to −0.7 V_{RHE} as in the operando X-ray absorption spectroscopy (XAS) experiment, conducted in 0.1 M $NaNO_3$ and 0.1 M NaOH at 25°C under nitrogen (Figure S1b). Within the applied potential range, nitrate ions (NO_3^-) were adsorbed and reduced. Although partial dissolution and reconstruction led to a slight decrease in Cu mass (2.10 mg cm^{-2}), these conditions were essential for correlating the operando XAS results with the electrochemical performance.

Material characterization.

X-ray diffraction (XRD) analysis was carried out using a SmartLab diffractometer (Rigaku, Japan) with Cu–K_α radiation operating at 40 kV and 30 mA. Surface morphology was analyzed by a field-emission scanning electron microscope (FE-SEM, JSM-IT800is, JEOL, Japan) equipped with an energy-dispersive X-ray spectrometer (EDS). Elemental mapping was conducted at low magnification (250×) in a large depth of focus (LDF) mode using a secondary electron detector (SED) at 15 kV, covering multiple regions to determine the average Cu/O ratio, excluding carbon signals.

Electrochemical measurement

Electrochemical experiments were performed in a commercial H-type two-compartment cell (VB12, EC Frontier, Japan), equipped with an acid trap (0.1 M H_2SO_4) and connected to an online gas chromatograph (490 MicroGC, Agilent Technologies, USA) (Figure S2a). The cathode compartment contained an Hg/HgO reference electrode for alkaline conditions (pH 13) along with the Cu_2O/C or Cu/C working electrode (1 × 1 cm^2). The anode compartment accommodated a coiled Pt wire counter electrode. The compartments were separated by an anion exchange membrane (Fumasep FAA-

3-50, 45-50 μm thick, Fumatech, Germany). The catholyte consisted of 20 mL of 0.1 M NaOH and 0.1 M $NaNO_3$, and the anolyte was 20 mL of 0.1 M NaOH (pH 12.9) unless otherwise noted. Argon (20 mL min^{-1}) was bubbled into the catholyte for 20 minutes before and purged in the headspace during the reaction. The experiments were conducted at room temperature (~25°C) with continuous stirring to enhance mass transport at the liquid–electrode interface.

Linear sweep voltammograms (J–V curves) were measured from +0.8 to −1.5 V_{RHE} at a scan rate of 0.01 V s^{-1}. Applied potentials were referenced to the RHE scale using the Nernst equation.

For alkaline conditions (pH 13): $E_{RHE} = E_{Hg/HgO} + 0.0591 \times pH + E^0_{Hg/HgO}$

where $E^0_{Hg/HgO}$ is +0.118 V_{SHE}.

Chronoamperometry (CA) was employed at fixed applied potentials for 1 h. The Faradaic efficiency (FE) of liquid products was calculated as:

$$\% FE = \frac{n \times e \times F}{J_{\text{recorded}} \times t} \times 100$$

where n is the amount of product (mol), e the number of electrons involved in the reaction (2 for NO_2^- or 8 for NH_4^+ formation), F the Faraday constant (96485 C mol^{-1}), $J_{recorded}$ the current density, and t the reaction time (s). The term $n \times e \times F$ represents the charge (C) from a product, and $J_{recorded} \times t$ is the total charge passed (C) during the reaction. For H_2 analysis, FE was calculated as:

$$\% FE = \frac{c \times e \times F}{J_{recorded}} \times 100$$

where c is hydrogen production rate (mol s^{-1}) and $c \times e \times F$ gives the corresponding current density (mA cm^{-2}) from detected hydrogen.

Operando quick X-ray absorption spectroscopy (XAS) experiment

Cu K-edge XAS (edge energy = 8.979 keV) measurement was performed at BL9A of the Photon Factory synchrotron (KEK, Tsukuba, Japan, Proposal No. 2025G640). A Si (111) monochromator was used for the selection of the energy of the incident X-ray. XAS spectra were acquired in transmission mode with ion chambers and collected in quick-scan mode.

For operando XAS, Cu_2O/C sample (2.25 mg cm^{-2}) was used as the working electrode (1 × 1 cm^2) in a custom-made H-type electrochemical cell (Figure S2b). The cathode compartment accommodated an Hg/HgO reference electrode. The anode compartment accommodated a coiled Pt wire counter electrode. Electrolyte solution (20 mL) was used as both the catholyte and the anolyte for the experiment. The two compartments were separated by Fumasep® FAA-3-50. Nitrogen gas was first bubbled (flow rate = 20 mL min^{-1}) into the catholyte for 10 min before measurements and then purged in the headspace during operando measurement. Electrochemical measurements were performed using an Ivium Vertex potentiostat. Chronoamperometry was conducted at potentials from 0.6 to −0.7 V_{RHE}, with each step maintained for 600 s (10 min). XAS spectra were collected simultaneously, starting 30 s after bias application, with seven spectra recorded in a duration of 600 s at each potential.

X-ray absorption near-edge structure (XANES) and extended X-ray absorption fine structure (EXAFS) analysis were performed using the Athena software. Linear combination analysis (LCA) of the XANES spectra was conducted using the

spectra at open circuit potential (OCP) as the Cu(I) reference and the last spectra at the most negative potential as the Cu(0) reference. The Cu(0) spectrum shape differed from that of the Cu foil, likely due to smaller crystalline sizes.

The EXAFS oscillation function *χ(k)* was made up of a wave vector k range of 0 to 15 Å^{-1}. Fourier transformation was performed on k^3-weighted *χ(k)* over k = 3–12 Å^{-1}. Curve fitting was carried out using the Artemis program in the k-range of 3 to 12 Å^{-1} for the spectra obtained by inverse Fourier transformation in the bond-length range of 1.5 to 2.8 Å for the first shell Cu−Cu coordination in metallic Cu. Fitting parameters included bond length (R), coordination number (N), Debye-Waller factor (σ^2), and the correction to the photoelectron reference energy value (ΔE_0). The amplitude reduction factor (S_0^2) was obtained from the Cu foil fitting. The R-factor, an indicator of the quality of EXAFS fitting, was lower than 0.2% for samples containing Cu(0) states. For samples containing the mixture of Cu(I) and Cu(0) oxidation states, the R-factor was higher due to the contribution of the Cu−Cu bond from the second coordination sphere, which was not included in the fit.

Product analysis

Ammonium ion (NH_4^+) was quantified using ion chromatography (Eco IC, Metrohm, Switzerland) with a Metrosep C 6–250/4.0 column and a 3.0 mM HNO_3 eluent. A calibration curve was made using NH_4Cl standard solutions. Nitrite ion (NO_2^-) was analyzed on the same instrument using a Metrosep A Supp 4–250/4.0 column. The eluent was a mixture of 3.2 mM Na_2CO_3 and 1.0 mM $NaHCO_3$. Regeneration of the suppressor was performed with 100 mM H_2SO_4, and ultrapure water was used for rinsing. A calibration curve was made using $NaNO_2$ standard solutions.

Gaseous products were analyzed using an online gas chromatograph (490 MicroGC, Agilent Technologies, USA) equipped with a thermal conductivity detector, a CP 4900BF MOD 10M MS column, and high-purity argon as the carrier gas.

Tables (Table S1–S6)

Table S1. Electrochemical NO_3RR to ammonia on selected copper-based electrodes in an H-type cell [1–14]

Electrocatalyst	% FE	Ammonia production rate / mmol h^{-1} cm^{-2}	Potential / V_{RHE}	Electrolyte	pH
Cu/Cu_2O nanowire array [1]	95.8	0.245	−0.85	0.5 M Na_2SO_4<br>200 ppm NO_3^-	Neutral
Cu(111) nanosheet/C fiber [2]	99.7	0.023*	−0.15	0.1 M KOH<br>10 mM KNO_3	Alkaline
Oxide-derived Cu [3]	92.0	1.1	−0.15	1 M KOH<br>0.1 M KNO_3	14
Ar plasma-treated Cu_2O/C fiber [4]	89.5	0.083*	−0.55*	0.5 M Na_2SO_4<br>50 ppm NO_3^-	Neutral
Cu-incorporated perylene film [5]	85.9	0.026*	−0.4	0.1 M PBS<br>500 ppm NO_3^-	7
Cu-Cu_2O/Ti foil [6]	92.0	0.28	−0.5	1 M KOH<br>0.1 M KNO_3	14
Reduced Cu_2O/Cu/C felt [7]	84.4	0.127*	−0.25	1 M KOH<br>250 ppm NO_3^-	14
Cu/2D-CuO_x nanocomposite [8]	97.6	0.087*	−0.8	0.1M NaOH<br>0.1 M Na_2SO_4<br>50 ppm NO_3^-	Alkaline
C/Cu derived from Cu_2O (111) [9]	~80.0	*n. a.*	−0.3	1 M NaOH<br>0.1 M $NaNO_3$	13.6
Zn-doped Cu nanosheets [10]	98.4	1.62×10^3	−0.85	0.5 M K_2SO_4<br>0.1 M KNO_3	Neutral
Cu/Cu_2O/Au-Ti coated Si wafer [11]	90–100	n.a.	−0.76*	0.5 M Na_2SO_4<br>0.05 M $NaNO_3$	5.8
Cu_2O nanocubes/C [12]	83–93	$42–48\times10^{-3}$	−0.30	0.1 M Na_2SO_4<br>n.a. mL NaOH<br>8 mM $NaNO_3$	12
Cu foil [13]	91.5	143.1×10^{-3}	−0.4	1M KOH<br>50 mM KNO_3	14
Cu_2O nanocubes/C [14]	~74.0	$\sim13\times10^{-3}$	−0.6	0.1 M Na_2SO_4<br>8 mM $NaNO_3$	7
Cu_2O/C fiber<br>(this work)	72.5	0.27	−0.7	0.1 M $NaNO_3$<br>0.1 M NaOH	13
Cu/C fiber<br>(this work)	89.7	0.30	−0.7	0.1 M $NaNO_3$<br>0.1 M NaOH	13

"*n.a.*" is not available information. *The value is converted from related articles.

Table S2. LCA results from XANES fitting under operando conditions with and without $NaNO_3$.

Applied potential / V_{RHE}	w/ $NaNO_3$			w/o $NaNO_3$		
	R-factor	LCA weight		R-factor	LCA weight	
		Cu(I)	Cu(0)		Cu(I)	Cu(0)
OCP	1.39E-05	1	0	1.75E-05	0.994	0.00608
0.6	1.81E-05	1	0	0.000416	0.951	0.0491
0.5	2.06E-05	1	0	0.000474	0.952	0.0480
0.4	2.75E-05	1	0	0.000184	0.966	0.0335
0.3	4.84E-05	0.975	0.0245	9.92E-05	0.949	0.0512
0.2	4.01E-05	0.962	0.0379	0.000116	0.840	0.160
0.1	0.000147	0.717	0.283	0.000388	0.0765	0.924
0	1.96E-05	0.525	0.475	0.000578	1.10E-08	1
−0.1	7.96E-05	0.354	0.645	0.000757	9.44E-09	1
−0.2	7.52E-05	0.194	0.806	0.000710	8.75E-09	1
−0.3	5.20E-06	0.0933	0.907	0.000392	2.62E-09	1
−0.4	5.80E-06	0.0433	0.957	*n.a.*	*n.a.*	*n.a.*
−0.5	5.10E-06	0.0303	0.969	*n.a.*	*n.a.*	*n.a.*
−0.6	5.21E-05	0.0153	0.985	*n.a.*	*n.a.*	*n.a.*
−0.7	3.40E-06	0.000331	1	*n.a.*	*n.a.*	*n.a.*

**n.a.* is "not available" due to poor fitting caused by disruption from H_2 bubble formation on the electrode.

For the LCA of the operando XANES data, *R*-factors were consistently low (typically 10^{-5} to 10^{-4}), indicating highly reliable spectral deconvolution and stable baseline behavior across potentials.

XAS spectra were collected simultaneously, starting 30 seconds after bias application, with seven spectra recorded over 600 seconds at each potential. The tables provided represent one representative EXAFS fitting result at each potential, selected from these replicates to reflect the typical structural behavior at that stage of reduction.

Table S3. EXAFS fitting under operando conditions with and without $NaNO_3$.

w/ $NaNO_3$					
**Applied potential / V_{RHE}**	**CN**	***R* / Å**	**d*E* / eV**	**D.W. / Å^2**	***R*-factor**
−0.7	10.5	2.54	2.50	0.00866	0.00213
−0.6	10.4	2.54	3.69	0.00859	0.00195
−0.5	10.3	2.54	3.83	0.00861	0.00188
−0.4	10.1	2.54	3.98	0.00869	0.00134
−0.3	9.42	2.54	4.02	0.00855	0.00261
−0.2	8.70	2.54	4.25	0.00878	0.00575
−0.1	7.19	2.54	4.19	0.00880	0.0228
0	5.30	2.55	4.38	0.00879	0.0814

w/o $NaNO_3$					
**Applied potential / V_{RHE}**	**CN**	***R* / Å**	**d*E* / eV**	**D.W. / Å^2**	***R*-factor**
−0.3	10.7	2.54	2.65	0.00888	0.00176
−0.2	10.7	2.54	2.51	0.00878	0.00210
−0.1	10.4	2.54	2.54	0.00868	0.00162
0	10.4	2.54	2.38	0.00893	0.00146

* EXAFS fitting parameters: $3 \le k \le 12$, $1.5 \le R \le 2.8$

For EXAFS fitting, *R*-factors ranged from 0.001 to 0.005 under most conditions, supporting the accuracy of the derived structural parameters. Slightly higher values (up to ~0.08) were observed at potentials where Cu_2O was partially reduced, likely due to increased local disorder or mixed-phase composition.

XAS spectra were collected simultaneously, starting 30 seconds after bias application, with seven spectra recorded over 600 seconds at each potential. The tables provided represent one representative EXAFS fitting result at each potential, selected from these replicates to reflect the typical structural behavior at that stage of reduction.

Table S4. Quantification of liquid products (NH_4^+ and NO_2^-) at different stages of ex situ electrochemical NO_3RR using Cu_2O/C catalyst in 0.1 M $NaNO_3$ (pH 13)

Ammonium (NH_4^+)

**Potential range / V_{RHE}**	**Analyte**	**NH_4^+ / mmol**	**NH_4^+ FE (%)**	**Total NH_4^+ / mmol**	**Total FE (%)**
0.3 to −0.7	Catholyte	0.0490	63.9	0.0505	65.9
	Anolyte	0.0004	0.490		
	Acidic trap	0.0011	1.47		
0.3 to −0.5	Catholyte	0.0230	51.0	0.0230	51.0
	Anolyte	*n.d.*	*n.d.*		
	Acidic trap	*n.d.*	*n.d.*		
0.3 to −0.3	Catholyte	0.0038	15.7	0.0038	15.7
	Anolyte	*n.d.*	*n.d.*		
	Acidic trap	*n.d.*	*n.d.*		
0.3 to −0.1	Catholyte	*n.d.*	*n.d.*	*n.d.*	*n.d.*
	Anolyte	*n.d.*	*n.d.*		
	Acidic trap	*n.d.*	*n.d.*		
0.3 to 0.1	Catholyte	*n.d.*	*n.d.*	*n.d.*	*n.d.*
	Anolyte	*n.d.*	*n.d.*		
	Acidic trap	*n.d.*	*n.d.*		
OCP to 0.4	Catholyte	*n.d.*	*n.d.*	*n.d.*	*n.d.*
	Anolyte	*n.d.*	*n.d.*		
	Acidic trap	*n.d.*	*n.d.*		

Nitrite (NO_2^-)

**Potential range / V_{RHE}**	**Analyte**	**NO_2^- / mmol**	**NO_2^- FE (%)**	**Total NO_2^- / mmol**	**Total FE (%)**
0.3 to −0.7	Catholyte	0.130	42.1	0.138	44.9
	Anolyte	0.0084	2.74		
0.3 to −0.5	Catholyte	0.0920	51.2	0.098	54.3
	Anolyte	0.0057	3.15		
0.3 to −0.3	Catholyte	0.0860	89.2	0.086	89.6
	Anolyte	0.0004	0.380		
0.3 to −0.1	Catholyte	0.0250	108	0.025	108
	Anolyte	*n.d.*	*n.d.*		
0.3 to 0.1	Catholyte	0.0011	53.2	0.001	53.2
	Anolyte	*n.d.*	*n.d.*		
OCP to 0.4	Catholyte	*n.d.*	*n.d.*	*n.d.*	*n.d.*
	Anolyte	*n.d.*	*n.d.*		

*Catholyte is 0.1 M $NaNO_3$ and 0.1 M NaOH; anolyte is 0.1 M NaOH; acidic trap employed 0.1 M H_2SO_4 as trapping agent of NH_4^+. *n.d.* is "not detected."

Low amounts of NH_4^+ are trapped in an acidic trap.[6] NH_4^+ and NO_2^- are transferred through the AEM membrane (cross-over) to the anolyte chamber.[15]

Table S5. Quantification of liquid products (NH_4^+ and NO_2^-) after electrochemical NO_3RR using Cu_2O/C electrode in 0.1 M $NaNO_3$ (pH 13) for 1 hour

Ammonium (NH_4^+)

**Potential / V_{RHE}**	**Sample**	**NH_4^+ / mmol**	**NH_4^+ FE (%)**	**Total NH_4^+ / mmol**	**Total FE (%)**
−0.7	Catholyte	0.210	67.3	0.226	72.5
	Anolyte	0.0100	3.28		
	Acidic trap	0.0059	1.93		
−0.5	Catholyte	0.120	57.0	0.128	61.1
	Anolyte	0.0060	2.92		
	Acidic trap	0.0024	1.17		
−0.3	Catholyte	0.0210	19.2	0.022	20.1
	Anolyte	0.0008	0.680		
	Acidic trap	0.0003	0.270		
−0.1	Catholyte	0.0015	3.06	0.002	3.06
	Anolyte	*n.d.*	*n.d.*		
	Acidic trap	*n.d.*	*n.d.*		
0.1	Catholyte	*n.d.*	*n.d.*	*n.d.*	*n.d.*
	Anolyte	*n.d.*	*n.d.*		
	Acidic trap	*n.d.*	*n.d.*		

Nitrite (NO_2^-)

**Potential / V_{RHE}**	**Sample**	**NO_2^- / mmol**	**NO_2^- FE (%)**	**Total NO_2^- / mmol**	**Total FE (%)**
−0.7	Catholyte	0.220	18.3	0.270	22.4
	Anolyte	0.0500	4.08		
−0.5	Catholyte	0.310	37.2	0.359	43.1
	Anolyte	0.0490	5.88		
−0.3	Catholyte	0.360	81.2	0.378	85.1
	Anolyte	0.0180	3.98		
−0.1	Catholyte	0.180	92.3	0.189	96.7
	Anolyte	0.0086	4.39		
0.1	Catholyte	0.0170	64.2	0.024	90.4
	Anolyte	0.0067	26.2		

*Catholyte is 0.1 M $NaNO_3$ and 0.1 M NaOH; anolyte is 0.1 M NaOH; acidic trap employed 0.1 M H_2SO_4 as the trapping agent of NH_4^+. *n.d.* is "not detected."

Low amounts of NH_4^+ are trapped on the acidic trap.[6] NH_4^+ and NO_2^- are transferred through the AEM membrane (cross-over) to the anolyte chamber.[15]

Table S6. Quantification of liquid products (NH_4^+ and NO_2^-) after electrochemical NO_3RR using reduced Cu/C electrode in 0.1 M $NaNO_3$ (pH 13) for 1 hour

Ammonium (NH_4^+)

**Potential / V_{RHE}**	**Sample**	**NH_4^+ / mmol**	**NH_4^+ FE (%)**	**Total NH_4^+ / mmol**	**Total FE (%)**
−0.7	Catholyte	0.270	79.9	0.303	89.7
	Anolyte	0.0230	6.90		
	Acidic trap	0.0095	2.85		
−0.5	Catholyte	0.240	80.6	0.266	89.4
	Anolyte	0.0200	6.78		
	Acidic trap	0.0062	2.08		
−0.3	Catholyte	0.140	68.1	0.150	72.8
	Anolyte	0.0068	3.31		
	Acidic trap	0.0029	1.40		
−0.1	Catholyte	0.0240	21.7	0.027	24.1
	Anolyte	0.0019	1.70		
	Acidic trap	0.0008	0.680		
0.1	Catholyte	0.0060	11.1	0.0068	12.4
	Anolyte	0.0008	1.38		
	Acidic trap	*n.d.*	*n.d.*		

Nitrite (NO_2^-)

**Potential / V_{RHE}**	**Sample**	**NO_2^- / mmol**	**NO_2^- FE (%)**	**Total NO_2^- / mmol**	**Total FE (%)**
−0.7	Catholyte	0.138	10.3	0.156	11.7
	Anolyte	0.0175	1.31		
−0.5	Catholyte	0.141	11.8	0.169	14.2
	Anolyte	0.0283	2.38		
−0.3	Catholyte	0.250	30.5	0.285	34.7
	Anolyte	0.0347	4.23		
−0.1	Catholyte	0.344	77.4	0.375	84.3
	Anolyte	0.0305	6.87		
0.1	Catholyte	0.180	82.3	0.199	90.9
	Anolyte	0.0188	8.60		

*Catholyte is 0.1 M $NaNO_3$ and 0.1 M NaOH; anolyte is 0.1 M NaOH; acidic trap employed 0.1 M H_2SO_4 as the trapping agent of NH_4^+. *n.d.* is "not detected."

Low amounts of NH_4^+ are trapped on the acidic trap.[6] NH_4^+ and NO_2^- are transferred through the AEM membrane (cross-over) to the anolyte chamber.[15]

Figures (Figures S1–S14)

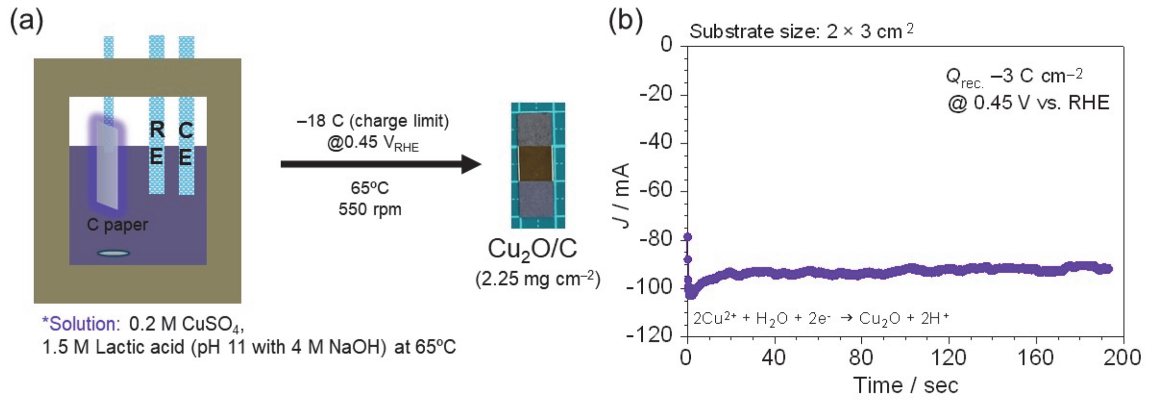

Figure S1. (a) Electrochemical cell setup and (b) current–time curve of Cu_2O electrodeposition.

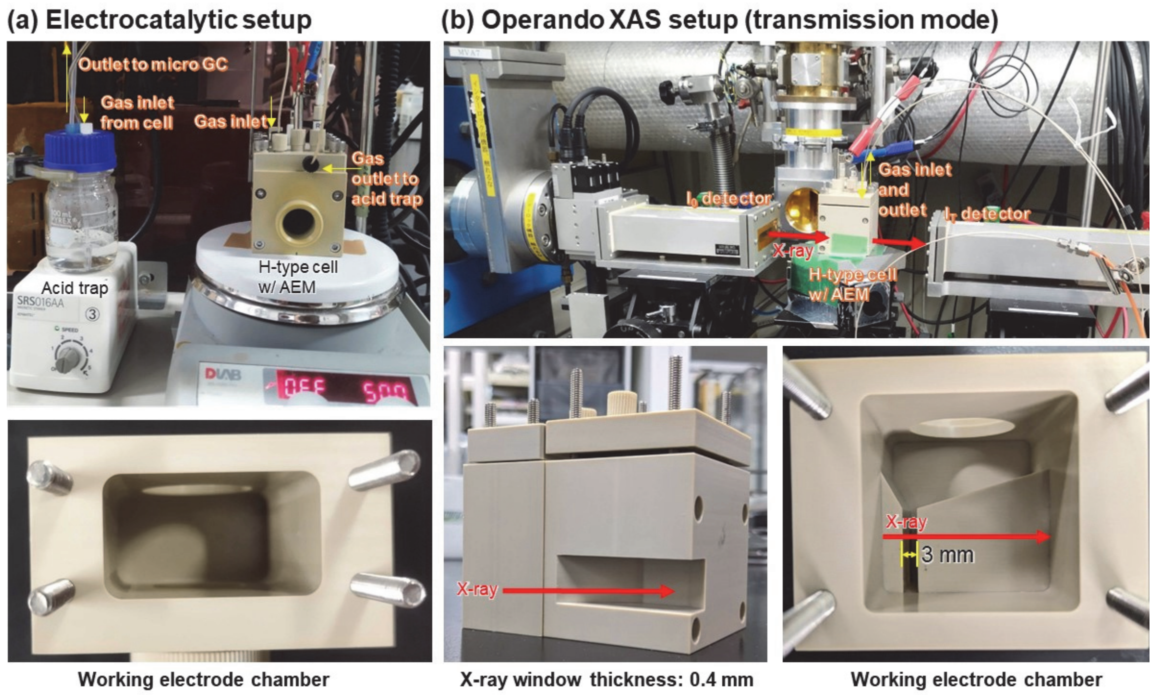

Figure S2 (a) Electrocatalytic setup and (b) operando XAS setup in the BL-9A beamline hutch (Photon Factory, Tsukuba, Japan).

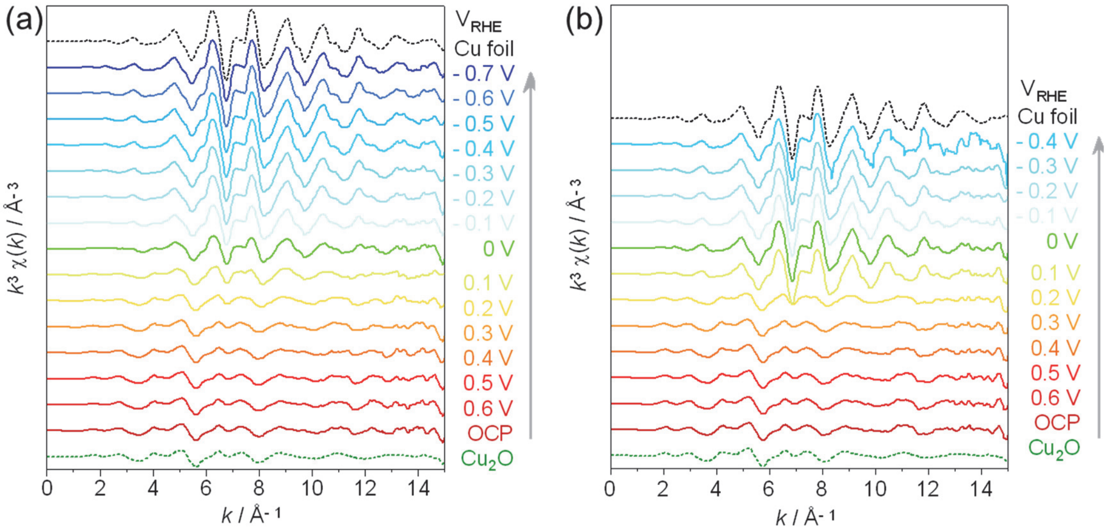

Figure S3. Operando EXAFS oscillations of Cu_2O/C during reactions in (a) 0.1 M $NaNO_3$ (pH 13) and (b) in 0.1 M NaOH (pH 13) without $NaNO_3$. The EXAFS oscillations were stacked vertically for clarity with an offset.

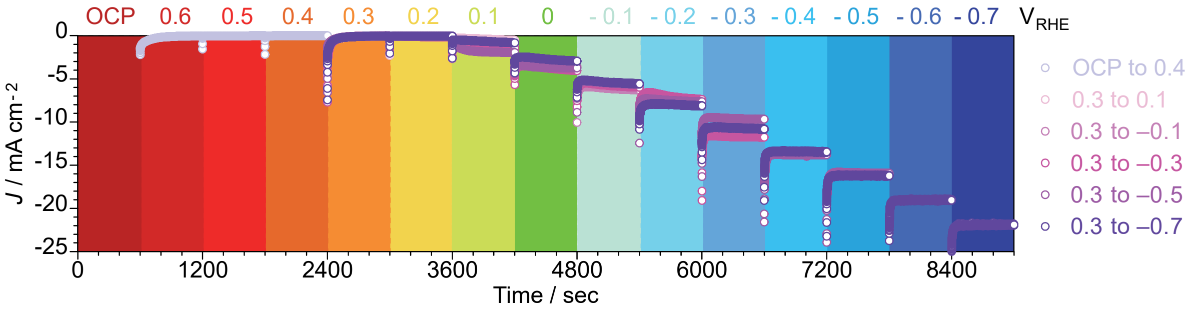

Figure S4. Current density profile of ex situ NO_3RR at different stages of the applied potential range using the operando XAS cell (Figure S2b).

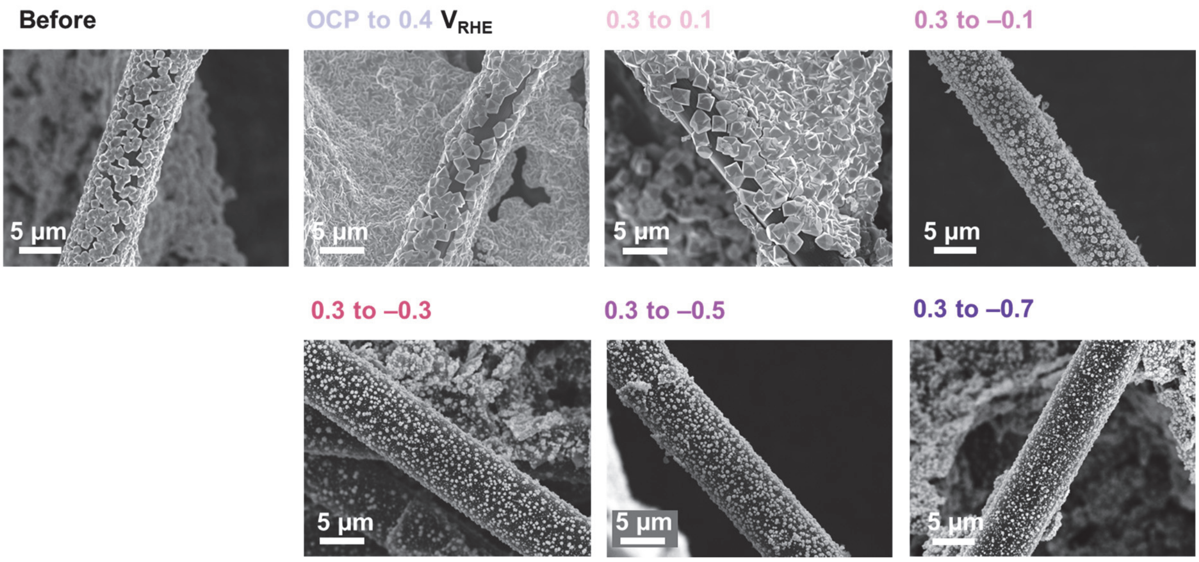

Figure S5. SEM images of Cu_2O/C before and after ex situ NO_3RR in 0.1 M $NaNO_3$ (pH 13) at different potential ranges.

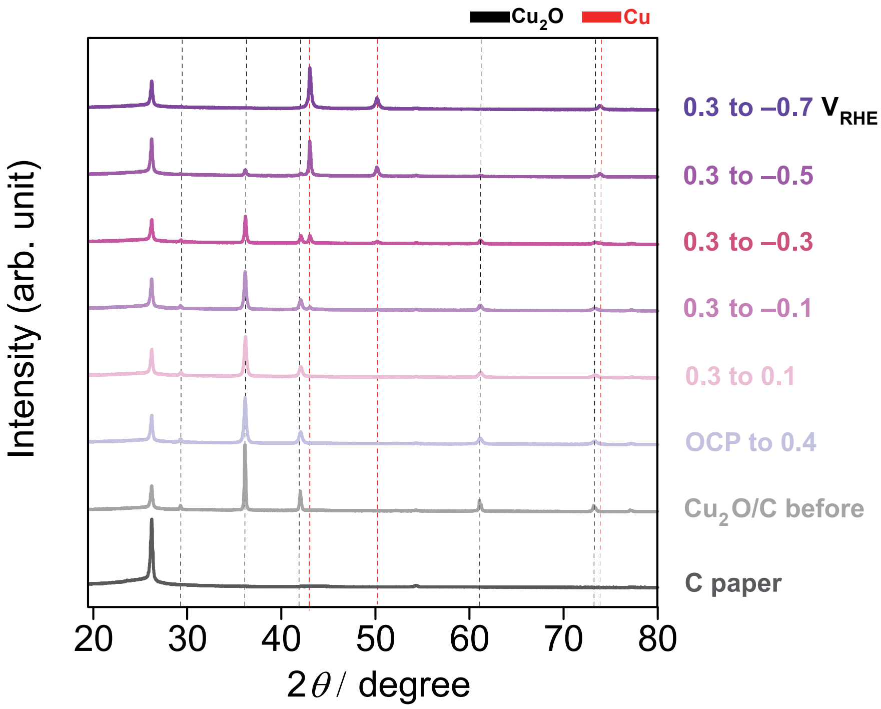

Figure S6. XRD patterns of Cu_2O/C before and after ex situ NO_3RR in 0.1 M $NaNO_3$ (pH 13) at different potential ranges.

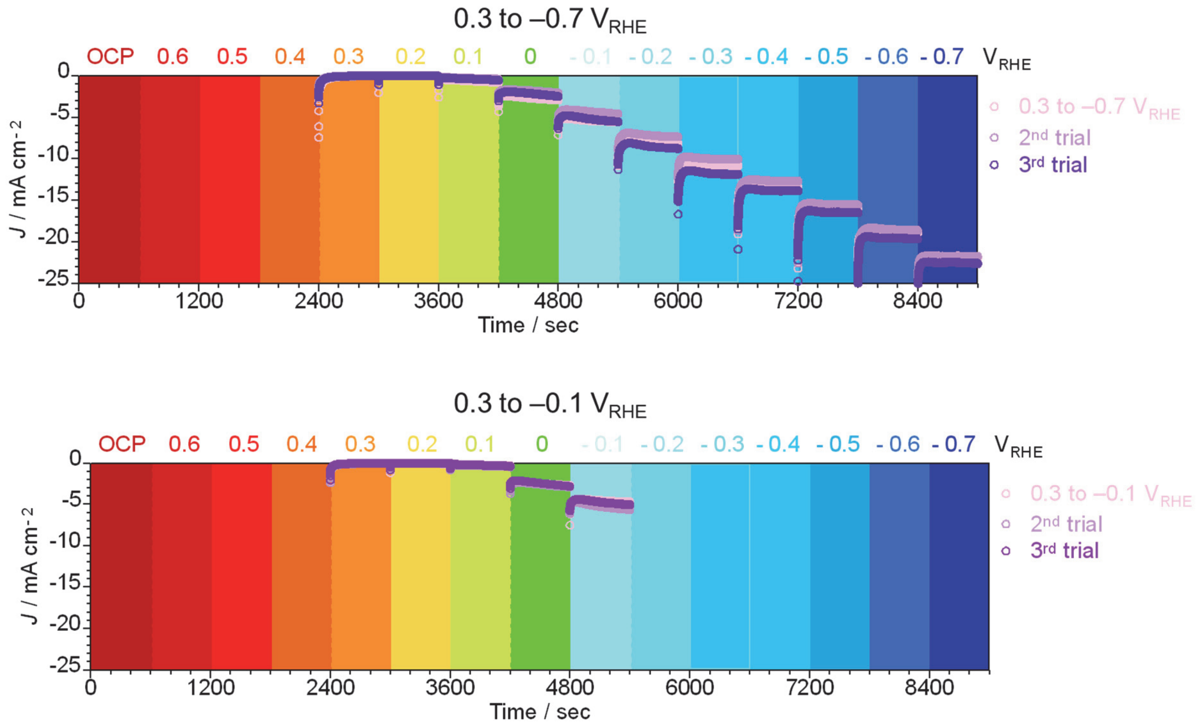

Figure S7. Current density profile of ex situ NO_3RR for NO_2^- and NH_4^+ quantifications over three independent trials.

At −0.7 V_{RHE}, the mean FE values were 65.8% for NH_4^+ (with a low standard deviation of 0.5%) and 41.2% for NO_2^- (with a higher standard deviation of 4.6%). Higher variability in NO_2^- (4.6–4.8%) may arise from desorption effects during the stepwise protocol, while NH_4^+ shows consistent quantification (0.5% variability).

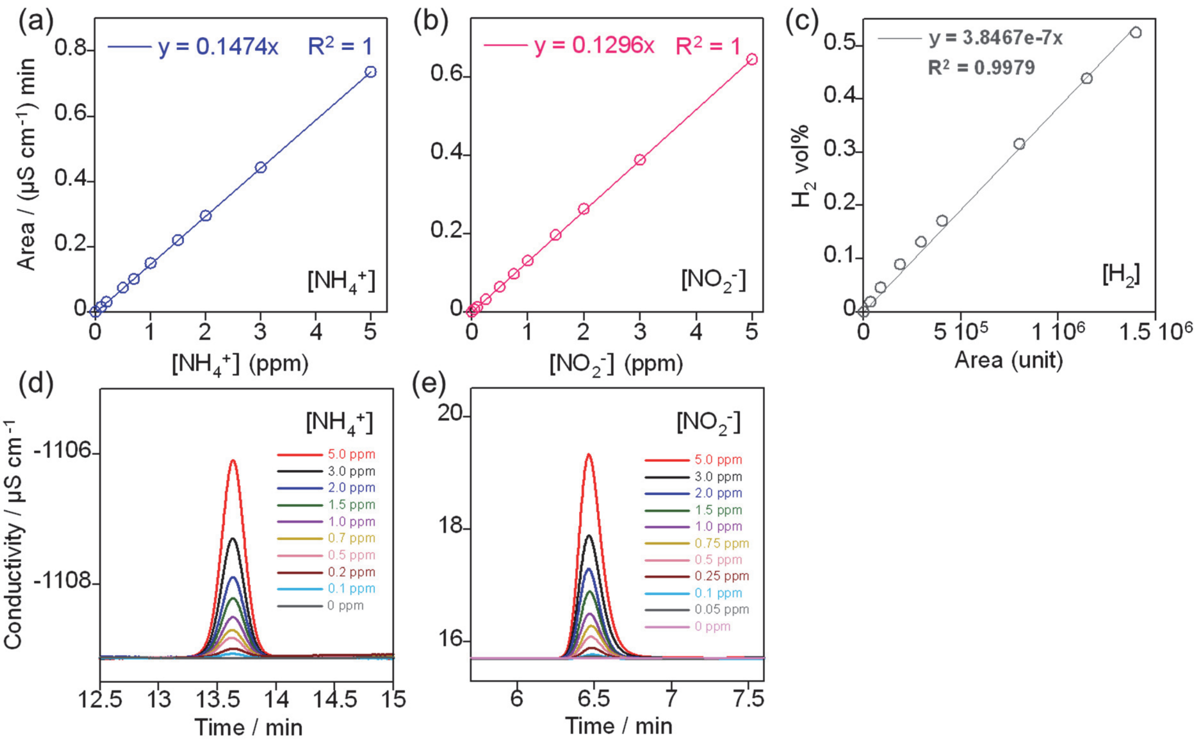

Figure S8. Calibration curves for the detection of (a) NH_4^+, (b) NO_2, and (c) H_2. Ion chromatograms for (d) NH_4^+ and (e) NO_2.

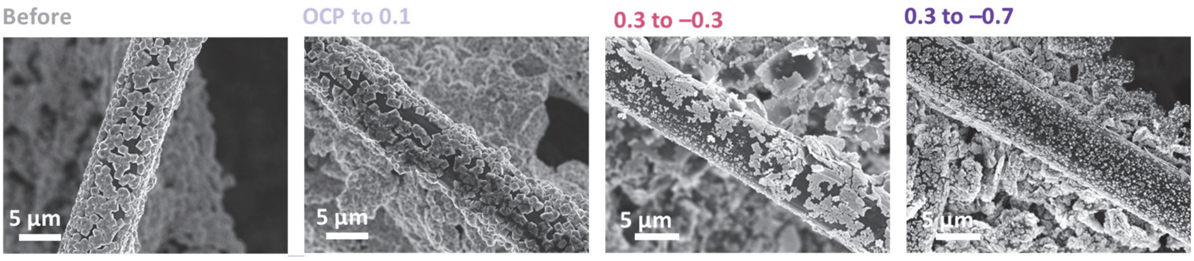

Figure S9. SEM–EDS images of Cu_2O/C before and after ex situ electrochemical reactions at different potential ranges in 0.1 M NaOH (pH 13) without $NaNO_3$.

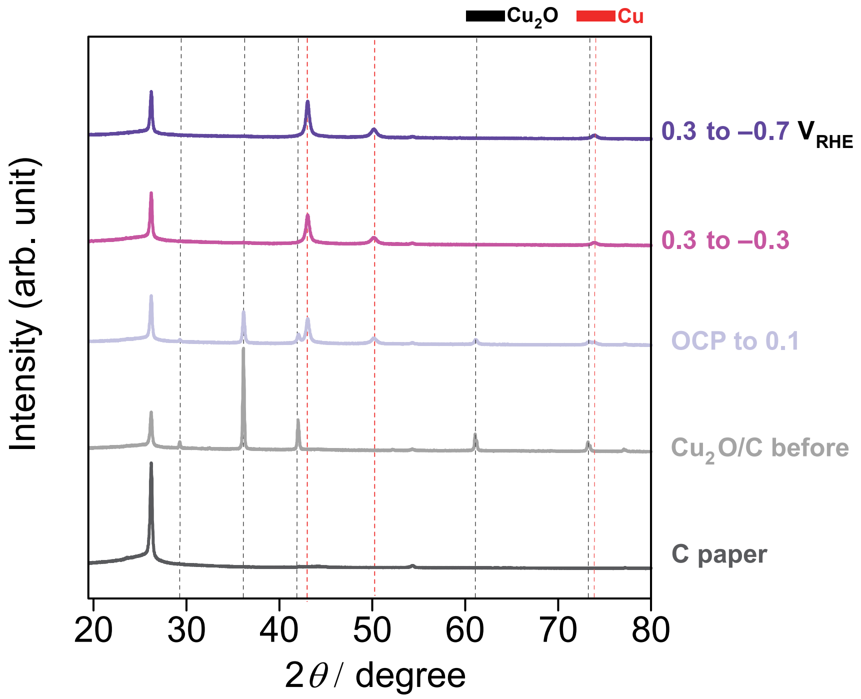

Figure S10. XRD patterns of Cu_2O/C before and after ex situ electrochemical reactions at different potential ranges in 0.1 M NaOH (pH 13) without $NaNO_3$.

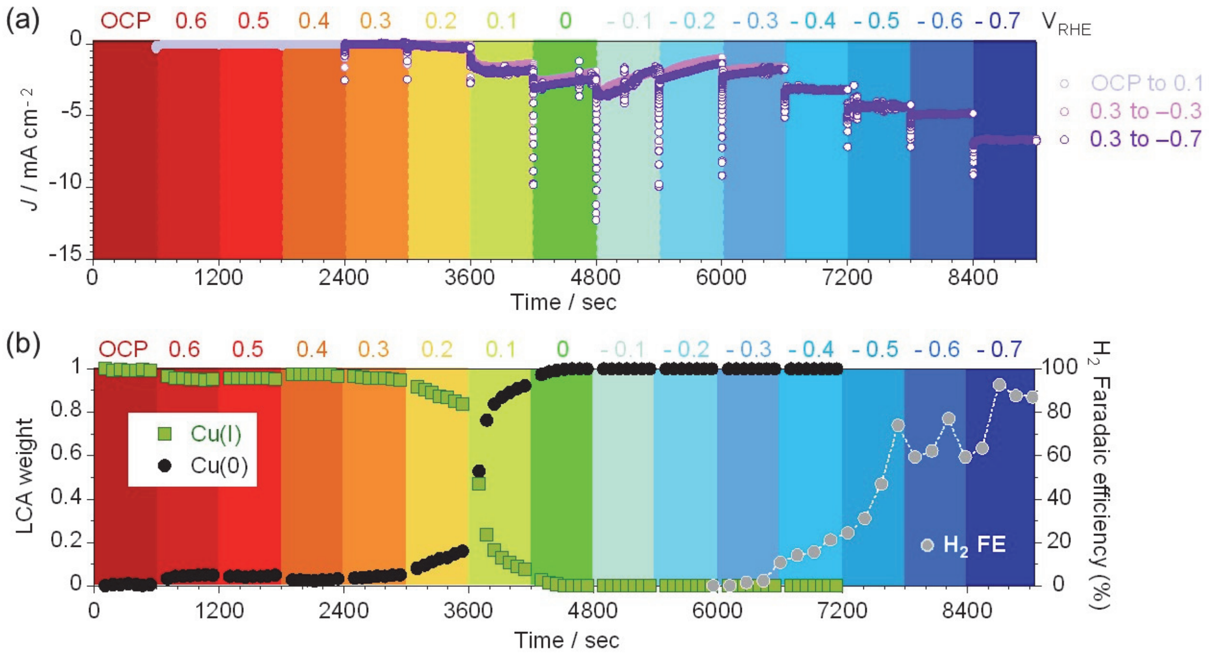

Figure S11. (a) Current density profile of Cu_2O/C during ex situ electrochemical reactions at various potential ranges in 0.1 M NaOH (pH 13) without $NaNO_3$. (b) LCA weights from in situ XANES spectra under operando conditions and H_2 FE from ex situ electrochemical reactions.

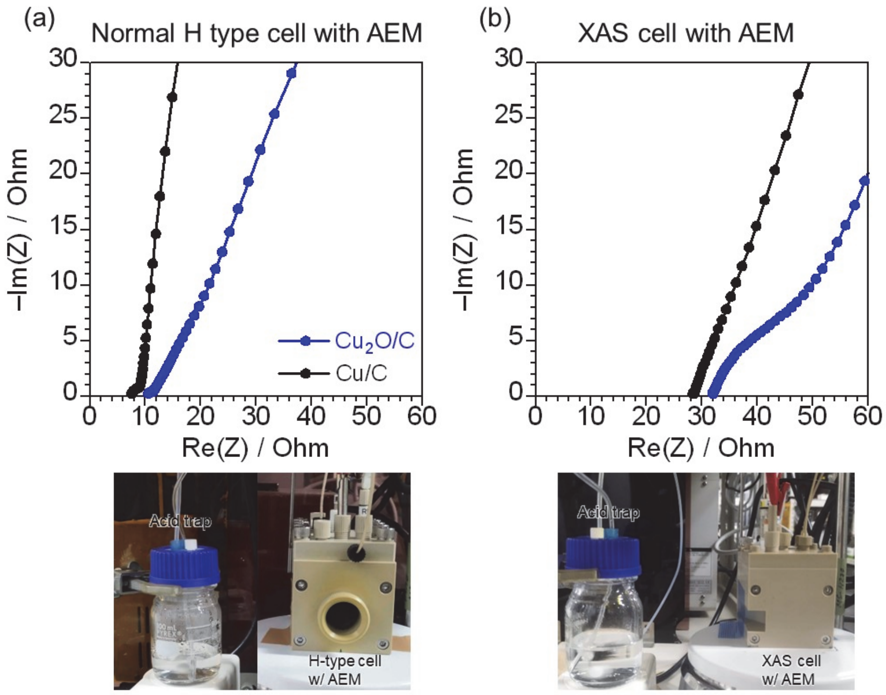

Figure S12. Nyquist plots of (a) the conventional H-type cell and (b) the operando XAS cell under alkaline conditions (pH 13, Catholyte: 0.1 M $NaNO_3$ + 0.1 M NaOH, Anolyte: 0.1 M NaOH).

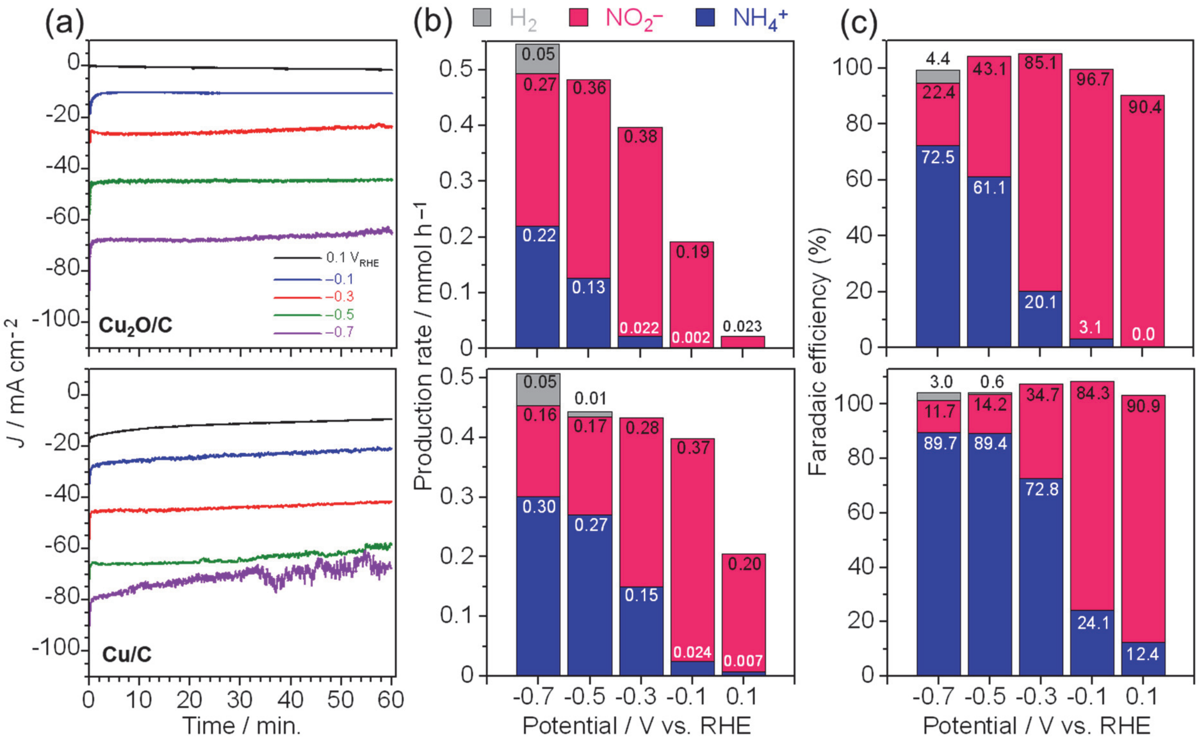

Figure S13. Electrocatalytic NO_3RR at constant potentials for 1 hour using of Cu_2O/C (top) and reduced Cu/C (bottom) electrodes: (a) current density–time plot, (b) production rates, and (c) product FEs.

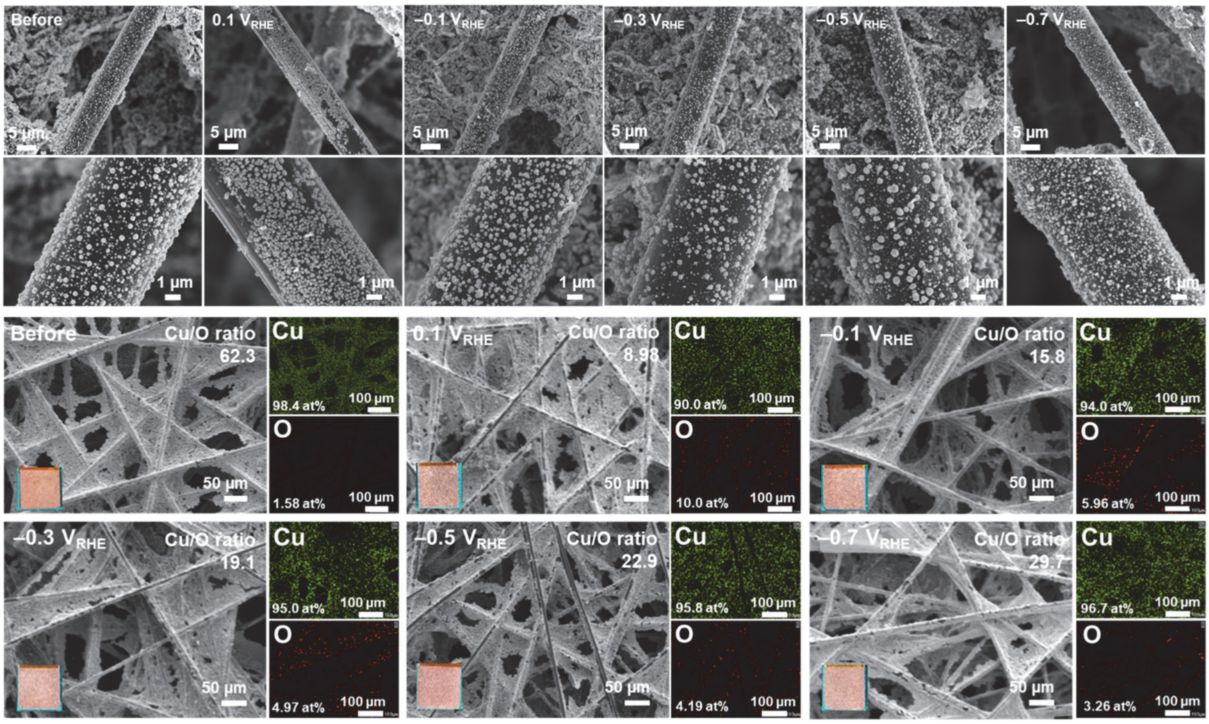

Figure S14. SEM–EDS images of reduced Cu/C electrodes before and after electrocatalytic NO_3RR at constant potentials for 1 hour.

References (Supporting information)

[1] Y. Wang, W. Zhou, R. Jia, Y. Yu, B. Zhang, *Angew. Chem., Int. Ed.* **2020**, *59*, 5350–5354.

[2] X. Fu, X. Zhao, X. Hu, K. He, Y. Yu, T. Li, Q. Tu, X. Qian, Q. Yue, M. R. Wasielewski, Y. Kang, *Appl. Mater. Today* **2020**, *19*, 100620.

[3] J. Yuan, Z. Xing, Y. Tang, C. Liu, *ACS Appl. Mater. Interfaces* **2021**, *13*, 52469–52478.

[4] Z. Gong, W. Zhong, Z. He, Q. Liu, H. Chen, D. Zhou, N. Zhang, X. Kang, Y. Chen, *Appl. Catal. B Environ.* **2022**, *305*, 121021.

[5] G.-F. Chen, Y. Yuan, H. Jiang, S.-Y. Ren, L.-X. Ding, L. Ma, T. Wu, J. Lu, H. Wang, *Nat. Energy* **2020**, *5*, 605–613.

[6] M. E. Chavez, M. Biset-Peiró, S. Murcia-López, J. R. Morante, *ACS Sustainable Chem. Eng.* **2023**, *11*, 3633–3643.

[7] W. Fu, Z. Hu, Y. Zheng, P. Su, Q. Zhang, Y. Jiao, M. Zhou, *Chem. Eng. J.* **2022**, *433*, 133680.

[8] H. Li, M. Tu, Y. Fang, T. Hao, B. Wang, *ACS Appl. Nano Mater.* **2023**, *6*, 18238–18246.

[9] D. Anastasiadou, Y. van Beek, W. Chen, T. Wissink, A. Parastaev, E. J. M. Hensen, M. Costa Figueiredo, *ChemCatChem* **2023**, *15*, 1–9.

[10] L. Wu, J. Feng, L. Zhang, S. Jia, X. Song, Q. Zhu, X. Kang, X. Xing, X. Sun, B. Han, *Angew. Chem., Int. Ed.* **2023**, *62*, e202307952.

[11] G. F. Costa, M. Winkler, T. Mariano, M. R. Pinto, I. Messias, J. B. Souza, I. T. Neckel, M. F. C. Santos, C. F. Tormena, N. Singh, R. Nagao, *Chem Catal.* **2024**, *4*, 100850.

[12] L. Bai, F. Franco, J. Timoshenko, C. Rettenmaier, F. Scholten, H. S. Jeon, A. Yoon, M. Rüscher, A. Herzog, F. T. Haase, S. Kühl, S. W. Chee, A. Bergmann, R. C. Beatriz, *J. Am. Chem. Soc.* **2024**, *146*, 9665–9678.

[13] Y. Kim, J. Ko, M. Shim, J. Park, H.-H. Shin, Z. H. Kim, Y. Jung, H. R. Byon, *Chem. Sci.* **2024**, *15*, 2578–2585.

[14] A. Yoon, L. Bai, F. Yang, F. Franco, C. Zhan, M. Rüscher, J. Timoshenko, C. Pratsch, S. Werner, H. S. Jeon, M. C. de O. Monteiro, S. W. Chee, B. Roldan Cuenya, *Nat. Mater.* **2025**, *24*, 762–769.

[15] P. Huang, H. Song, J. Yoo, D. A. Chipoco Haro, H. M. Lee, A. J. Medford, M. C. Hatzell, *Adv. Energy Mater.* **2024**, *14*, 2304202.
